# Nationwide in-hospital mortality and morbidity analysis of COVID-19 in advanced chronic kidney disease, dialysis and kidney transplant recipients

**DOI:** 10.3389/fmed.2023.1250631

**Published:** 2023-11-02

**Authors:** Mingyue He, Yichen Wang, Si Li, Avrum Gillespie

**Affiliations:** ^1^Department of Internal Medicine, Temple University Hospital, Philadelphia, PA, United States; ^2^Department of Medicine, Perelman School of Medicine, University of Pennsylvania, Philadelphia, PA, United States; ^3^Section of Nephrology, Hypertension and Kidney Transplantation, Lewis Katz School of Medicine at Temple University, Philadelphia, PA, United States

**Keywords:** Nationwide Inpatient Sample, COVID-19, advanced chronic kidney disease, dialysis, ESKD, kidney transplant recipient, in-hospital mortality, morbidity

## Abstract

**Background:**

Patients with advanced chronic kidney disease (CKD), end-stage kidney disease (ESKD), and kidney transplants (KT) are at an elevated risk for COVID-19 infection, hospitalization, and mortality. A comprehensive comparison of morbidity and mortality between these populations with kidney disease and individuals without any kidney disease is lacking.

**Methods:**

We analysed the 2020 Nationwide Inpatient Sample (NIS) database for non-elective adult COVID-19 hospitalizations, categorizing patients into advanced CKD, ESKD, KT, and kidney disease-free cohorts. Our analysis included a description of the distribution of comorbidities across the entire spectrum of CKD, ESKD, and KT. Additionally, we investigated in-hospital mortality, morbidity, and resource utilization, adjusting for potential confounders through multivariable regression models.

**Results:**

The study included 1,018,915 adults hospitalized for COVID-19 in 2020. The incidence of advanced CKD, ESKD, and KT in this cohort was 5.8%, 3.8%, and 0.4%, respectively. Patients with advanced CKD, ESKD, and KT exhibited higher multimorbidity burdens, with 90.3%, 91.0%, and 75.2% of patients in each group having a Charlson comorbidity index (CCI) equal to or greater than 3. The all-cause in-hospital mortality ranged from 9.3% in kidney disease-free patients to 20.6% in advanced CKD, 19.4% in ESKD, and 12.4% in KT patients. After adjusting for potential confounders at both the patient and hospital levels, CKD stages 3–5; ESKD; and KT were found to be associated with increased odds of mortality, with adjusted odds ratios (aOR) of 1.34, 1.80, 2.66, 1.97, and 1.69, respectively.

**Conclusion:**

Patients hospitalized for COVID-19 with advanced CKD, ESKD, or KT demonstrated a higher burden of comorbidities and increased mortality rates compared to those without kidney disease. After adjusting for confounders, CKD stages 3–5; ESKD; and KT were identified as independent risk factors for in-hospital mortality, illustrating a dose-response relationship between the odds of mortality and adverse outcomes as CKD progressed from stages 3 to 5. Our study highlights the necessity for enhanced management of comorbidities, targeted interventions, and vigorous vaccination efforts to mitigate the risk of adverse outcomes in the vulnerable populations of patients with CKD, ESKD, and KT.

## 1. Introduction

The coronavirus disease 2019 (COVID-19), caused by the severe acute respiratory syndrome coronavirus-2 (SARS-CoV-2), was initially identified in China and rapidly escalated into a global pandemic by March 2020, exerting substantial strain on healthcare systems worldwide (1). Subsequent investigations confirmed its association with significant mortality, particularly among individuals with pre-existing health conditions (2–14). Identified factors that increase the risk of COVID-19-related hospitalization and mortality include advanced age, male gender, Black or Asian ethnicities, socioeconomic disadvantage, and certain comorbidities such as obesity, diabetes, chronic pulmonary or cardiovascular conditions, chronic liver diseases, and malignancies (2–5). Notably, individuals with advanced chronic kidney disease (CKD) and end stage kidney disease (ESKD) on maintenance dialysis have a pronounced vulnerability to COVID-19, as well as a tendency towards worse clinical outcomes in the context of COVID-19 (5–10). Furthermore, the immunosuppressed state of kidney transplant (KT) recipients has been identified as a potential risk factor for severe COVID-19 and elevated mortality (9–14).

Despite the abundance of research, there is a paucity of comprehensive national-level data from the United States that compares in-hospital mortality and clinical outcomes of COVID-19 among patients with advanced CKD (stages 3–5), ESKD, and KT, against a control group of individuals without any underlying kidney conditions. Previous investigations often encountered methodological limitations, such as insufficient sample sizes, suboptimal data collection, a lack of rigorous stratification and controls, or an oversight of relevant comorbidities and socioeconomic factors. Although meta-analyses have attempted to overcome these limitations, they have been hampered by the very same methodological challenges, making cross-study comparisons difficult. Utilizing aggregated outcomes from studies without adequate consideration or adjustment for potential confounders can lead to constrained conclusions, a fact that has been well recognized in literature (8, 15). This challenge is further exacerbated by significant heterogeneity arising from amalgamation of data from diverse global sources.

To address this knowledge gap, we employed the Nationwide Inpatient Sample (NIS)—the largest inpatient database in the United States, to evaluate the in-hospital mortality and morbidity of patients admitted with COVID-19 in 2020, differentiating between those with and without pre-existing kidney conditions. The primary objective of our investigative initiative is to elucidate the clinical outcomes of individuals with CKD, ESKD, and KT hospitalized due to COVID-19, juxtaposed against a control cohort without any pre-existing kidney disease. To the best of our knowledge, this endeavor represents the first nationwide, population-based comparative analysis that delves into the clinical outcomes of COVID-19 hospitalizations, stratified by kidney disease, in the United States. We posit that the findings from our research will provide healthcare practitioners with an enriched perspective on how CKD/ESKD/KT affects the clinical trajectory and outcomes of COVID-19.

## 2. Methods

### 2.1. Data source

This study is a retrospective, observational, population-based analysis leveraging the 2020 NIS database, a part of the Healthcare Cost and Utilization Project (HCUP), supported by the Agency for Healthcare Research and Quality (AHRQ) (16). The NIS is the largest publicly available, all-payer inpatient database in the U.S., and draws from billing records of hospitals nationwide. Updated annually, it encompasses a full calendar year of clinical and resource data on both the patient and hospital levels. The patient-level data includes primary and secondary diagnoses (coded using the International Classification of Diseases, 10th revision, Clinical Modification (ICD-10-CM) coding system), procedures, admission and discharge status, demographic details, and utility sources. The hospital-level data are categorized by factors such as ownership/control, bed capacity, teaching status, and geography. The NIS’s design ensures a stratified representation of non-federal acute care hospitals across the U.S., pulling data from 48 states and the district of Columbia, covering 98% of the U.S. population and 97% of community hospital discharges. Through its systemic sampling methodology, the NIS captures about 20% of discharges from U.S. hospitals, resulting in a dataset of 32,355,827 hospitalizations for the calendar year of 2020. Each discharge is then weighted to ensure that the NIS is nationally representative (weight = total number of discharges from all acute care hospitals in the United States/number of discharges included in the 20% sample).

All data for this study are publicly available at https://hcup-us.ahrq.gov/db/nation/nis/nisdbdocumentation.jsp.

### 2.2. Ethics statement

Approval from an Institutional Review Board was not required for this study due to its retrospective nature and the use of de-identified data. The study was conducted in strict adherence to the data-use agreements of the NIS-HCUP.

### 2.3. Study subjects

We employed the 2020 NIS database to identify all patients hospitalized with a primary diagnosis of COVID-19. We excluded patients under the age of 18 and those admitted to the hospital for elective reasons, as opposed to emergency admissions. Following these exclusions, the remaining patients in the study cohort were stratified based on their pre-existing kidney disease status into five distinct categories: the advanced CKD group (comprising patients with a secondary diagnosis of CKD stages 3, 4, or 5); the ESKD group (encompassing those with a secondary diagnosis of ESKD or those dependent on dialysis); the KT group (patients with a secondary diagnosis indicating a history of kidney transplantation); the kidney disease-free group (consisting of patients without any secondary diagnosis of kidney disease) and others (patients who do not fit into any of the aforementioned categories). Data extraction was performed using the ICD-10-CM diagnostic codes (refer to Supplementary Table S1).

### 2.4. Study variables

The primary outcome of interest in this study was all-cause in-hospital mortality. The secondary outcomes include (1) morbidity, ascertained by the incidence rates of septic shock, acute respiratory failure, acute respiratory distress syndrome (ARDS), mechanical ventilation, and vasopressor requirement; and (2) resource utilization, evaluated by the length of hospital stay (LOS) and total hospitalization charges.

To mitigate the impact of confounders, potential confounding variables were extracted and considered. These variables included patient demographics like age, gender, and race; socioeconomic factors such as household income and insurance status (primary payer); prevalent comorbidities such as diabetes, hypertension, obesity, congestive heart failure, coronary artery disease, chronic pulmonary disease, chronic liver disease, non-metastatic solid tumors, and metastatic cancer. Furthermore, variables such as history of alcohol consumption, smoking, drug abuse, and hospital characteristics (including bed capacity, teaching affiliation, and geographical region) were taken into account. The Charlson comorbidity index (CCI) was adopted to measure the cumulative impact of comorbidities. This index offers a validated, efficient, and readily applicable means to estimate the mortality risk associated with coexisting diseases (17). A comprehensive list of specific diagnostic codes used in this process is detailed in Supplementary Table S1.

### 2.5. Statistical analysis

Statistical analyses were performed using Stata 17.0 (StataCorp LLC, College Station, TX, United States). The NIS employs a multifaceted sampling design incorporating stratification, clustering, and weighting. Consequently, data were processed as survey data and adjusted using the discharge-level weight variable provided by AHRQ. Unless explicitly mentioned, all statistics presented herein reflect these weighted estimates. Categorical variables were presented as percentages, while continuous variables as means with standard deviations. The chi-square test and ANOVA were used for descriptive comparisons.

Univariate regression analyses were conducted to ascertain the unadjusted odds ratio for primary and secondary outcomes with respect to potential confounding factors. If data were absent for any of the regression variables, the corresponding patient entry was omitted from the analysis. These confounders were subsequently accounted for in subsequent multivariable regression models. To verify the absence of multicollinearity in our multivariable models, we assessed the variance inflation factor (VIF). It is worth noting that only the Charlson comorbidity index (CCI) presented a VIF greater than five and was therefore excluded from subsequent multivariable analysis. None of the remaining risk factors manifested a VIF above five, thereby confirming the absence of multicollinearity. A refined regression model was formulated, encompassing only those variables that exhibited a potential association (*p* < 0.2) in the univariable regression analysis. All statistical interpretations were grounded on a significance level defined by a *p*-value threshold of 0.05.

## 3. Results

### 3.1. Patient and hospital characteristics

**Figure 1:**
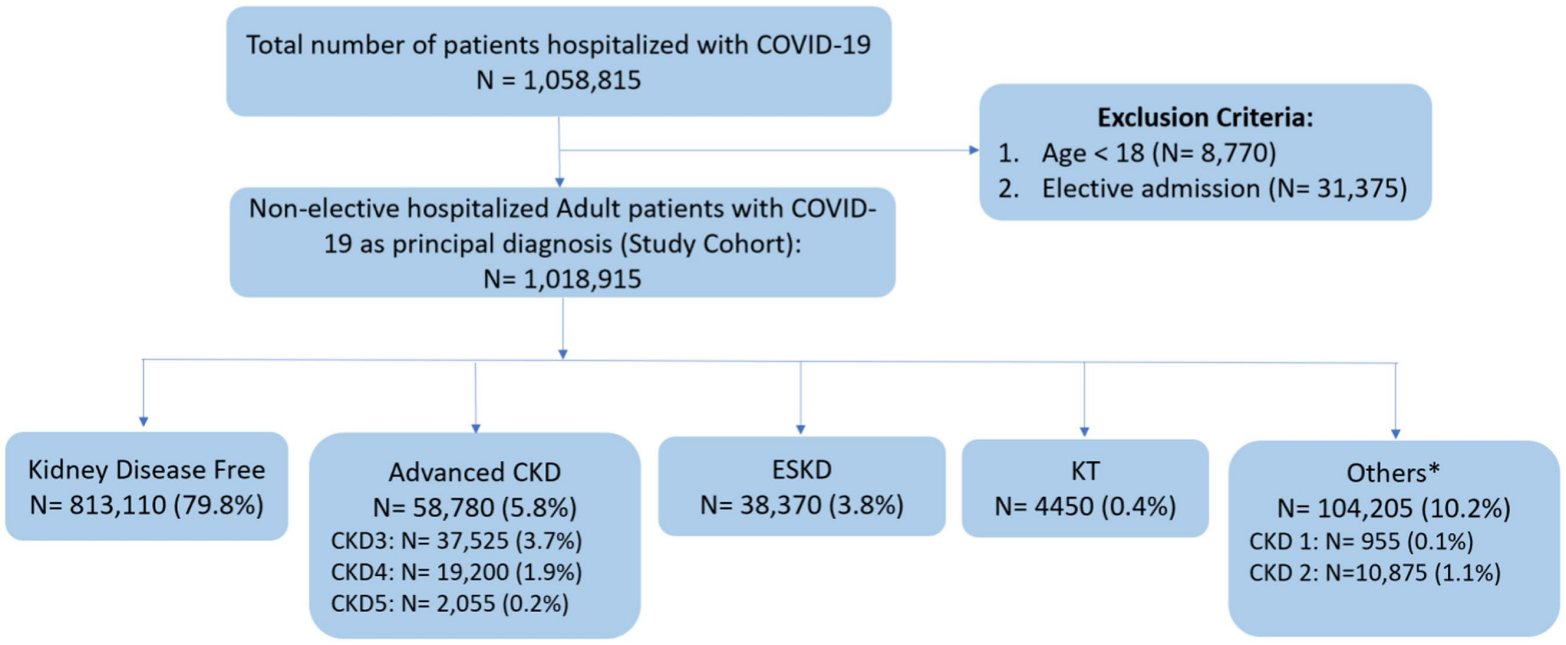
Flow diagram of study selection. COVID-19, coronavirus disease 2019; CKD, chronic kidney disease; ESKD, end stage kidney disease; KT, kidney transplant. ^*^Others: include patients with a diagnosis of CKD stage 1, CKD stage 2, and unspecified CKD stages.

The flow diagram illustrating the study’s inclusion process is depicted in Figure 1. The study cohort included 1,018,915 adult patients non-electively admitted for COVID-19 in 2020. Within this group, 58,780 (5.8%) patients had advanced CKD: 37,525 (3.7%) were at CKD stage 3, 19,200 (1.9%) at CKD stage 4, and 2,055 (0.2%) at CKD stage 5. Additionally, 38,370 (3.8%) had ESKD, 4,450 (0.4%) patients were KT recipients, and 813,110 (79.8%) had no documented underlying kidney conditions.

Table 1 delineates the patient-level and hospital-level characteristics, with Supplementary Table S2 providing additional details. The cohort had an average age of 64.7 ± 0.1 years, and 47.2% were female. White patients accounted for the majority of hospital admissions at 52.4%, followed by Hispanics at 20.6% and Blacks at 18.6%. Geographically, the southern region recorded the highest number of COVID-19 hospitalizations, representing 41.5% of the cases (for specific regions of hospitals in NIS, please refer to Supplementary Table S3). In terms of payment, Medicare was the primary payer for 54.9% of admissions. Regarding comorbidities, hypertension was the most prevalent, afflicting 67.7% of patients, followed by diabetes at 40.8%. Approximately one-quarter of the patients were either obese, had chronic pulmonary disease, or had a history of smoking. Notably, 28.1% of patients had a Charlson comorbidity index (CCI) of 3 or higher.

**Table 1:** Baseline characteristics of adult patients hospitalized for COVID-19.

		Total *N* = 1,018,915	Kidney disease-free *N* = 813,110 (79.8%)	Advanced CKD *N* = 58,780 (5.8%)	ESKD *N* = 38,370 (3.8%)	KT *N* = 4,450 (0.4%)
Age (year)		64.7 ± 0.1	62.8 ± 0.1	74.3 ± 0.1	64.0 ± 0.2	59.1 ± 0.4
Female sex *n* (%)		480,540 (47.2%)	390,900 (48.1%)	27,865 (46.6%)	17,120 (43.6%)	1,580 (35.5%)
**Race *n* (%)**						
	White	518,470 (52.4%)	411,590 (52.2%)	31,090 (53.5%)	11,315 (29.6%)	1,695 (39.2%)
	Black	183,825 (18.6%)	134,005 (17.0%)	16,075 (27.2%)	13,190 (34.4%)	1,130 (26.1%)
	Hispanic	204,095 (20.6%)	174,425 (22.1%)	7,495 (12.6%)	9,880 (25.8%)	1,135 (26.2%)
	Asian or Pacific Islander	32,370 (3.3%)	26,480 (3.4%)	1,725 (2.9%)	1,530 (4.0%)	145 (3.4%)
	Native American	10,160 (1.0%)	8,230 (1.0%)	355 (0.6%)	850 (2.3%)	70 (1.6%)
	Others	40,210 (4.1%)	34,090 (4.3%)	1,815 (3.1%)	1,535 (4.0%)	150 (3.5%)
**Median household income for patient’s zip code *n* (%)**						
	$ 1–$ 49,999	339,825 (33.9%)	267,995 (33.5%)	21,080 (35.5%)	16,245 (42.4%)	1,550 (35.2%)
	$ 50,000–$ 64,999	276,400 (27.6%)	220,260 (27.5%)	15,690 (26.5%)	10,235 (26.5%)	1,200 (27.3%)
	$ 65,000–$ 85,999	222,140 (22.2%)	178,000 (22.3%)	12,680 (21.3%)	7,545 (19.5%)	910 (20.7%)
	$ 86,000 or more	164,555 (16.4%)	133,830 (16.7%)	9,840 (16.7%)	4,520 (11.7%)	740 (16.8%)
**Insurance status *n* (%)**						
	Medicare	531,325 (54.9%)	377,985 (49.3%)	45,410 (77.9%)	28,430 (74.2%)	2,935 (67.9%)
	Medicaid	119,350 (12.3%)	103,300 (13.5%)	4,330 (7.4%)	4,795 (12.6%)	400 (9.3%)
	Private including HMO	281,625 (29.1%)	253,565 (33.1%)	8,000 (13.5%)	4,560 (11.8%)	940 (21.7%)
	Self-pay	34,875 (3.6%)	32,375 (4.2%)	750 (1.3%)	505 (1.3%)	50 (1.2%)
**Comorbidities *n* (%)**						
	Congestive heart failure	171,070 (16.8%)	93,150 (11.5%)	24,810 (41.4%)	17,275 (44.2%)	960 (21.6%)
	Coronary artery disease	187,390 (18.4%)	117,755 (14.5%)	20,805 (34.7%)	13,270 (34.1%)	1,030 (23.2%)
	Chronic pulmonary disease	239,610 (23.5%)	184,315 (22.7%)	16,645 (28.0%)	8,445 (21.6%)	495 (11.1%)
	Diabetes	415,645 (40.8%)	293,805 (36.1%)	35,975 (59.6%)	27,980 (71.6%)	2,490 (56.0%)
	Hypertension	690,155 (67.7%)	502,070 (61.8%)	55,565 (92.4%)	37,375 (95.2%)	3,855 (86.6%)
	Solid tumor without metastasis	20,390 (2.0%)	15,375 (1.9%)	1,425 (2.4%)	650 (1.7%)	65 (1.5%)
	Metastatic cancer	8,010 (0.8%)	6,210 (0.8%)	510 (0.9%)	245 (0.6%)	30 (0.7%)
	Chronic liver disease	46,480 (4.6%)	36,335 (4.5%)	2,745 (4.6%)	2,535 (6.5%)	265 (6.0%)
	Obesity	280,870 (27.6%)	230,195 (28.3%)	14,760 (24.6%)	8,770 (22.5%)	810 (18.2%)
	Alcohol use	19,325 (1.9%)	16,305 (2.0%)	830 (1.4%)	495 (1.3%)	35 (0.8%)
	Smoking	223,720 (22.0%)	170,990 (21.0%)	15,055 (25.2%)	8,190 (20.8%)	950 (21.4%)
	Drug use	18,730 (1.8%)	15,550 (1.9%)	935 (1.5%)	705 (1.8%)	45 (1.0%)
**Charlson comorbidity index (CCI) *n* (%)**						
	0	282,400 (27.7%)	282,400 (34.7%)	0 (0%)	0 (0%)	0 (0%)
	1	283,960 (27.9%)	283,960 (34.9%)	0 (0%)	0 (0%)	0 (0%)
	2	166,160 (16.3%)	142,915 (17.6%)	5,905 (9.7%)	3,590 (9.0%)	1,105 (24.8%)
	≥3	286,395 (28.1%)	103,835 (12.8%)	54,215 (90.3%)	35,705 (91.0%)	3,345 (75.2%)
**Hospital bed size *n* (%)**						
	Small	256,360 (25.2%)	206,495 (25.4%)	14,475 (24.1%)	8,020 (20.4%)	825 (18.5%)
	Medium	295,700 (29.0%)	237,465 (29.2%)	17,225 (28.6%)	11,485 (29.2%)	1,165 (26.2%)
	Large	466,855 (45.8%)	369,150 (45.4%)	28,420 (47.3%)	19,790 (50.4%)	2,460 (55.3%)
**Hospital location/teaching status *n* (%)**						
	Rural	110,395 (10.8%)	89,060 (11.0%)	5,500 (9.2%)	2,300 (5.8%)	330 (7.4%)
	Urban nonteaching	196,855 (19.3%)	159,240 (19.6%)	10,695 (17.8%)	7,025 (18.0%)	560 (12.6%)
	Urban teaching	711,665 (69.9%)	564,810 (69.5%)	43,925 (73.0%)	29,970 (76.2%)	3,560 (80.0%)
**Hospital region *n* (%)** ^a^						
	Northeast	183,195 (18.0%)	146,355 (18.0%)	11,460 (19.0%)	7,060 (18.0%)	890 (20.0%)
	Midwest	236,315 (23.2%)	180,185 (22.2%)	15,050 (24.9%)	8,180 (20.9%)	1,120 (25.2%)
	South	422,431 (41.5%)	341,346 (42.0%)	25,685 (42.9%)	16,410 (41.7%)	1,645 (37.0%)
	West	176,974 (17.4%)	145,225 (17.9%)	7,925 (13.2%)	7,645 (19.5%)	795 (17.9%)

a Regions of hospital in NIS: please refer to Supplementary Table S3.

Distinct variations in patient-level and hospital-level characteristics were evident upon stratification by different kidney disease statuses, all of which demonstrated statistical significance, as detailed in Supplementary Table S2.

The advanced CKD group predominantly consisted of older patients with a mean age of 74.3 ± 0.1 years and exhibited a heightened prevalence of comorbidities. For example, 90.3% of these patients had a CCI of 3 or higher, compared to only 12.8% in the group without kidney disease. Additionally, 41.4% of the advanced CKD group had congestive heart failure, which was significantly higher than the 11.5% in the kidney disease-free group. Other prominent comorbidities in the advanced CKD group included coronary artery disease (34.7%), diabetes (59.6%), and hypertension (92.4%), all of which were significantly higher compared to the kidney disease-free group (14.5%, 36.1%, and 61.8%, respectively).

The ESKD group had a significant proportion of patients who identified as Black, 34.4%, compared to 17.0% in the kidney disease-free group. Additionally, the prevalence of low annual income was markedly higher among the ESKD cohort, with 42.4% earning an annual income below $50,000, compared to 33.5% in the kidney disease-free group, 35.5% in the advanced CKD group, and 35.2% in the KT group. Clinically, the ESKD group had the highest prevalence of comorbid conditions such as congestive heart failure at 44.2%, diabetes at 71.6%, hypertension at 95.2%, and 91% with a CCI ≥3.

The KT group was the youngest demographic with an average age of 59.1 ± 0.4 years. This group also had the lowest proportion of female patients (35.5%). From a comorbidity perspective, a significant 75.2% of the KT group had a CCI equal to or exceeding 3, which stands in notable contrast to the 12.8% observed in the kidney disease-free group. Additionally, 80% of patients within the KT group were treated in urban teaching institutions.

A comparison of patient and hospital characteristics across all stages of CKD was also conducted. All observed variations were found to be statistically significant, as detailed in Supplementary Table S4.

### 3.2. In-hospital all-cause mortality

Among the 1,018,915 adult patients non-electively admitted for COVID-19, 113,180 patients died in the hospital, yielding an overall in-hospital all-cause mortality rate of 11.1%. The mortality rate stratified by kidney disease is shown in Table 2. Patients with advanced CKD had the highest in-hospital mortality rate, followed by those in the ESKD category. Specifically, the mortality rates were as follows: kidney disease-free at 9.3%, advanced CKD at 20.6%, ESKD at 19.4%, and KT at 12.4% (*p* < 0.001).

**Table 2:** Outcomes of adult patients hospitalized for COVID-19 stratified by kidney disease.

		Total	Kidney disease-free	Advanced CKD	ESKD	KT	*p*-value
Mortality	All cause in hospital mortality *N* %	113,180 (11.1%)	75,270 (9.3%)	12,100 (20.6%)	7,445 (19.4%)	550 (12.4%)	<0.001
**Morbidity**							
	Septic shock *N* %	34,365 (3.4%)	24,450 (3.0%)	2,775 (4.7%)	3,075 (8.0%)	205 (4.6%)	<0.001
	Acute respiratory failure *N* %	575,370 (56.5%)	458,645 (56.4%)	32,970 (56.1%)	21,025 (54.8%)	2,065 (46.4%)	<0.001
	ARDS *N* %	53,560 (5.3%)	41,640 (5.1%)	3,705 (6.3%)	2,430 (6.3%)	275 (6.2%)	<0.001
	Mechanical ventilation N %	87,295 (8.6%)	63,640 (7.8%)	6,735 (11.5%)	4,895 (12.8%)	355 (8.0%)	<0.001
	Vasopressor N %	18,475 (1.8%)	13,240 (1.6%)	1,560 (2.6%)	1,550 (4.0%)	80 (1.8%)	<0.001
**Resource utilization**							
	Mean LOS (days)	7.5 ± 0.03	7.2 ± 0.04	9.0 ± 0.09	10.3 ± 0.15	7.0 ± 0.25	<0.001
	Mean total hospitalization charges (USD)	79,101 ± 1,144	76,511 ± 1,168	87,470 ± 1,648	127,376 ± 3,099	81,760 ± 4,588	<0.001

ARDS, acute respiratory distress syndrome; LOS, length of hospital stay.

Using univariate logistic regression analyses, we analyzed potential confounding factors to determine the unadjusted odds ratio for mortality. The smoking factor was excluded from subsequent multivariable regression models due to a *p*-value greater than 0.2 in univariable regression. After adjusting for relevant confounders, the advanced CKD, ESKD, and KT groups all demonstrated significantly increased odds of in-hospital mortality compared to the kidney disease-free group. Specifically, the adjusted OR (aOR) of mortality for advanced CKD was 1.52 [95% confidence interval (CI): 1.44–1.61, *p* < 0.001], for ESKD was 1.97 (95% CI: 1.83–2.12, *p* < 0.001), and for KT was 1.69 (95% CI: 1.36–2.09, *p* < 0.001) (refer to Table 3).

**Table 3:** Outcomes of univariate and multivariable regression adjusted analysis stratified by kidney disease.

		Kidney disease-free	Advanced CKD	ESKD	KT
In hospital mortality	OR (95% CI)	1.00	2.54 (2.42–2.68)	2.36 (2.22–2.51)	1.38 (1.12–1.70)
	*p* value		<0.001	<0.001	0.002
	aOR (95% CI)	1.00	**1.52 (1.44–1.61)**	**1.97 (1.83–2.12)**	**1.69 (1.36–2.09)**
	*p*-value		**<0.001**	**<0.001**	**<0.001**
Septic shock	OR (95% CI)	1.00	1.60 (1.45–1.76)	2.81 (2.56–3.08)	1.56 (1.13–2.15)
	*p*-value		<0.001	<0.001	0.007
	aOR (95% CI)	1.00	**1.30 (1.17–1.45)**	**2.12 (1.90–2.37)**	**1.42 (1.01–1.99)**
	*p*-value		**<0.001**	**<0.001**	**0.042**
Acute respiratory failure	OR (95% CI)	1.00	0.99 (0.95–1.03)	0.94 (0.89–0.98)	0.67 (0.59–0.77)
	*p*-value		0.529	0.007	<0.001
	aOR (95% CI)	1.00	**0.92 (0.88–0.96)**	**0.94 (0.89–0.99)**	**0.72 (0.62–0.83)**
	*p*-value		**<0.001**	**0.014**	**<0.001**
ARDS	OR (95% CI)	1.00	1.25 (1.15–1.35)	1.25 (1.13–1.39)	1.22 (0.92–1.61)
	*p*-value		<0.001	<0.001	0.159
	aOR (95% CI)	1.00	**1.17 (1.07–1.28)**	1.07 (0.96–1.20)	1.19 (0.89–1.58)
	*p*-value		**<0.001**	0.233	0.235
Mechanical ventilation	OR (95% CI)	1.00	1.52 (1.43–1.62)	1.72 (1.60–1.85)	1.02 (0.80–1.30)
	*p*-value		<0.001	<0.001	0.865
	aOR (95% CI)	1.00	**1.11 (1.04–1.19)**	**1.29 (1.19–1.40)**	1.02 (0.80–1.30)
	*p*-value		**0.002**	**<0.001**	0.886
Vasopressor	OR (95% CI)	1.00	1.59 (1.41–1.79)	2.51 (2.20–2.86)	1.11 (0.68–1.79)
	*p*-value		<0.001	<0.001	0.681
	aOR (95% CI)	1.00	**1.31 (1.15–1.49)**	**1.96 (1.68–2.28)**	1.01 (0.62–1.66)
	*p*-value		**<0.001**	**<0.001**	0.957
LOS	*β* (95% CI)	Ref	1.74 (1.57–1.92)	3.10 (2.82–3.39)	−0.19 (−0.69 to 0.30)
	*p*-value		<0.001	<0.001	0.446
	Adjusted *β* (95% CI)	Ref	**0.88 (0.70–1.06)**	**2.31 (2.01–2.61)**	−0.18 (−0.69 to 0.33)
	*p*-value		**<0.001**	**<0.001**	0.492
Total hospitalization charges	*β* (95% CI)	Ref	10,958 (8,171–13,745)	50,865 (45,548-56,182)	5,249 (−3,499 to 13,997)
	*p*-value		<0.001	<0.001	0.240
	Adjusted *β* (95% CI)	Ref	**4,849 (1,960–7,738)**	**37,268 (31,938-42,598)**	2,952 (−5,862 to 11,767)
	*p*-value		**0.001**	**<0.001**	0.511

ARDS, acute respiratory distress syndrome; LOS, length of hospital stay; CI, confidence interval; aOR, adjusted odds ratio. Significant values (*p* < 0.05) are shown in bold.

Several independent risk factors associated with increased odds of in-hospital all-cause mortality were identified by the multivariable regression model (as detailed in Table 4). These factors encompassed age (aOR 1.05, 95% CI: 1.05–1.05, *p* < 0.001), obesity (aOR 1.22, 95% CI: 1.17–1.27, *p* < 0.001), diabetes (aOR 1.17, 95% CI: 1.13–1.21, *p* < 0.001), chronic heart failure (aOR 1.50, 95% CI: 1.44–1.56, *p* < 0.001), chronic lung disease (aOR 1.09, 95% CI: 1.05–1.13, *p* < 0.001), chronic liver disease (aOR 2.56, 95% CI: 2.39–2.73, *p* < 0.001), solid tumor without metastasis (aOR 1.16, 95% CI: 1.05–1.28, *p* < 0.001) and metastasis (aOR 1.74, 95% CI: 1.48–2.04, *p* < 0.001). Of note, CKD stage 5 and liver disease had an aOR of more than 2.5 for mortality. Female sex was associated with a decreased risk of mortality (aOR 0.7, 95% CI: 0.68–0.72, *p* < 0.001).

**Table 4:** Independent risk factors of mortality in patients admitted with COVID-19.

	Univariate regression model		Multivariate regression model	
	OR (95% CI)	*p*-value	OR (95%)	*p*-value
Age	1.05 (1.04–1.05)	<0.001	**1.05 (1.05–1.05)**	**<0.001**
Female sex	0.78 (0.76–0.80)	<0.001	**0.70 (0.68–0.72)**	**<0.001**
Race (White as reference)	B: 0.83 (0.79–0.88)	<0.001	0.99 (0.93–1.04)	0.610
	H: 0.89 (0.84–0.94)	<0.001	**1.26 (1.19–1.33)**	**<0.001**
	AP: 0.91 (0.82–1.01)	0.081	**1.13 (1.02–1.25)**	**0.024**
	NA: 1.33 (1.13–1.56)	0.001	**2.07 (1.73–2.48)**	**<0.001**
Median household income (first quartile as reference)	2nd: 0.94 (0.90–0.98)	0.005	**0.90 (0.85–0.94)**	**<0.001**
	3rd: 0.89 (0.85–0.93)	<0.001	**0.81 (0.76–0.85)**	**<0.001**
	4th: 0.92 (0.86–0.98)	0.006	**0.77 (0.72–0.82)**	**<0.001**
Primary payer (medicare as reference)	Medicaid: 0.46 (0.44–0.49)	<0.001	1.06 (0.99–1.13)	0.112
	Private: 0.37 (0.35–0.39)	<0.001	**0.89 (0.84–0.94)**	**<0.001**
	Self-pay: 0.41 (0.36–0.47)	<0.001	1.08 (0.93–1.24)	0.316
Obesity	0.79 (0.76–0.82)	<0.001	**1.22 (1.17–1.27)**	**<0.001**
Hypertension	1.59 (1.53–1.65)	<0.001	**0.84 (0.80–0.87)**	**<0.001**
Diabetes	1.36 (1.32–1.40)	<0.001	**1.17 (1.13–1.21)**	**<0.001**
Coronary artery disease	1.75 (1.70–1.81)	<0.001	0.97 (0.93–1.01)	0.134
Chronic heart failure	2.36 (2.28–2.44)	<0.001	**1.50 (1.44–1.56)**	**<0.001**
Chronic lung disease	1.23 (1.19–1.27)	<0.001	**1.09 (1.05–1.13)**	**<0.001**
Chronic liver disease	2.10 (1.99–2.23)	<0.001	**2.56 (2.39–2.73)**	**<0.001**
Chronic kidney disease (compared to no kidney disease)	CKD1: 1.68 (1.14–2.49)	0.010	1.41 (0.92–2.16)	0.111
	CKD2: 1.60 (1.40–1.83)	<0.001	1.10 (0.95–1.28)	0.192
	CKD3: 2.22 (2.08–2.37)	<0.001	**1.34 (1.25–1.44)**	**<0.001**
	CKD4: 3.11 (2.88–3.36)	<0.001	**1.80 (1.66–1.97)**	**<0.001**
	CKD5: 3.55 (2.84–4.42)	<0.001	**2.66 (2.08–3.40)**	**<0.001**
	ESKD: 2.36 (2.22–2.51)	<0.001	**1.97 (1.83–2.12)**	**<0.001**
	KT: 1.38 (1.12–1.70)	0.002	**1.69 (1.36–2.09)**	**<0.001**
Solid tumor without metastasis	1.72 (1.58–1.87)	<0.001	**1.16 (1.05–1.28)**	**0.004**
Metastasis	2.17 (1.92–2.46)	<0.001	**1.74 (1.48–2.04)**	**<0.001**
Alcohol	1.07 (0.97–1.18)	0.164	0.97 (0.87–1.08)	0.579
Smoking	0.98 (0.94–1.02)	0.228	N/A	N/A
Drug use	0.61 (0.54–0.69)	<0.001	**0.83 (0.72–0.95)**	**0.009**
Hospital bed size (small as reference)	Medium: 1.17 (1.09–1.25)	<0.001	**1.15 (1.08–1.24)**	**<0.001**
	Large: 1.15 (1.08–1.22)	<0.001	**1.19 (1.12–1.27)**	**<0.001**
Hospital region (NE as reference)	MW: 0.67 (0.62–0.73)	<0.001	**0.63 (0.58–0.68)**	**<0.001**
	S: 0.66 (0.61–0.72)	<0.001	**0.70 (0.65–0.76)**	**<0.001**
	W: 0.71 (0.66–0.78)	<0.001	**0.73 (0.67–0.80)**	**<0.001**
Hospital location/teaching (rural as reference)	Urban-nonteaching 1.06 (0.97–1.15)	0.182	**1.15 (1.05–1.26)**	**0.002**
	Urban teaching 1.25 (1.17–1.35)	<0.001	**1.34 (1.24–1.45)**	**<0.001**

B, Black; H, Hispanic; AP, Asian or Pacific Islander; NA, Native American; NE, Northeast; MW, Midwest; S, South; W, West; ARDS, acute respiratory distress syndrome; LOS, length of hospital stay. Significant values (*p* < 0.05) are shown in bold.

Hispanic, Asian/Pacific Islander and Native American patients had increased odds of mortality compared to White patients, whereas the mortality odds for Black patients were found to be comparable to those of White patients. Notably, Native American patients demonstrated the most pronounced increase in mortality odds, approximately double that of White patients, representing the highest mortality odds observed across all races and ethnicities. Additionally, patients from higher median household income brackets had lower mortality odds compared to those in the low-income bracket. Hospitals with medium and large bed capacities were associated with higher mortality when compared with small hospitals. Similarly, urban non-teaching and urban teaching hospitals were associated with higher mortality when compared with rural hospitals. Hospitals in other geographical locations had lower mortality when compared with hospitals in the northeast region.

### 3.3. Mortality outcome by CKD stages

We conducted a comprehensive analysis to assess all-cause in-hospital mortality and morbidity stratified by the different stages of CKD, as detailed in Supplementary Table S5. Additionally, we performed both univariate and multivariable logistic regression analyses, accounting for the various CKD stages; the results of these are presented in Supplementary Table S6.

**Figure 2:**
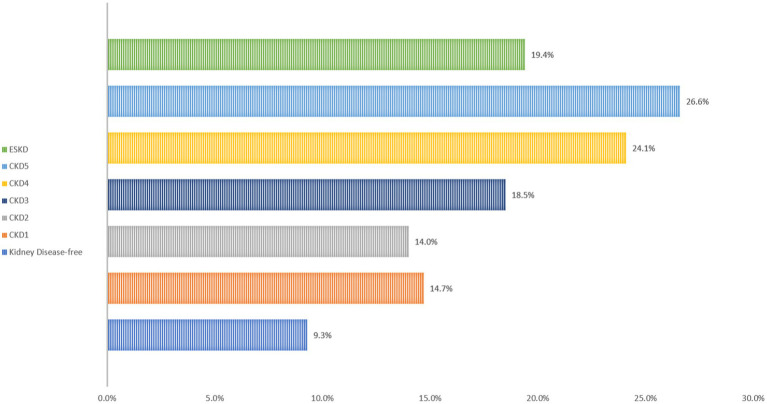
All cause in hospital mortality stratified by CKD stages.

For adult patients hospitalized due to COVID-19, the overall in-hospital mortality rates varied based on their CKD status. Specifically, patients without kidney disease exhibited a mortality rate of 9.3%. In contrast, those with CKD stage 1 had a rate of 14.7%, 14.0% for CKD stage 2, 18.5% for CKD stage 3, 24.1% for CKD stage 4, and 26.6% for CKD stage 5. Meanwhile, the mortality rate for patients with ESKD was 19.4%. These observed differences in mortality rates are significant, as illustrated in Figure 2 and further elucidated in Supplementary Table S5.

**Figure 3:**
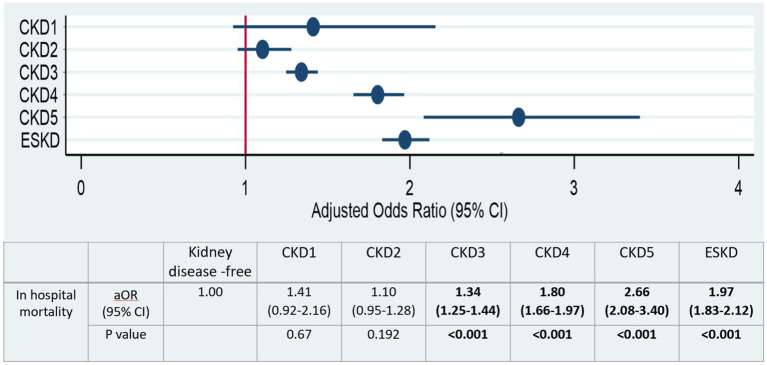
Adjusted odd ratios of all causes in hospital mortality stratified by CKD stages. Significant values (*p* < 0.05) are shown in bold.

Figure 3 and Supplementary Table S6 display the adjusted odds ratios (aOR) derived from the multivariable logistic regression analysis across different CKD stages. CKD stage 3 (aOR 1.34, 95% CI: 1.25–1.44, *p* < 0.001), CKD stage 4 (aOR 1.80, 95% CI: 1.66–1.97, *p* < 0.001), CKD stage 5 (aOR 2.66, 95% CI: 2.08–3.4, *p* < 0.001), and ESKD (aOR 1.97, 95% CI: 1.83–2.12, *p* < 0.001) exhibited a significant increase in all-cause in-hospital mortality odds when compared to patients without any kidney disease. Importantly, our data revealed a dose-response trend from CKD stage 3 to 5, with CKD 5 having the highest aOR among all variables investigated, including ESKD.

### 3.4. Morbidity

#### 3.4.1. Septic shock

The overall incidence of septic shock among hospitalized adult patients with COVID-19 was 3.4%. The incidence of septic shock in the kidney disease-free group, advanced CKD group, ESKD group, and KT group was 3.0%, 4.7%, 8.0%, and 4.6%, respectively (Table 2).

Factors such as hypertension, coronary artery disease, solid tumors without metastasis, metastasis, drug use, and chronic lung disease were excluded from subsequent multivariable regression models because their *p*-values were greater than 0.2 in the univariate regression. This adjusted logistic regression analysis revealed significantly increased odds for septic shock in advanced CKD, ESKD and KT groups when compared to the kidney disease-free group (aOR 1.30, 95% CI: 1.17–1.45, *p* < 0.001 for advanced CKD; aOR 2.12, 95% CI: 1.90–2.37, *p* < 0.001 for ESKD, aOR: 1.42, 95% CI: 1.01–1.99, *p* = 0.042 for KT) (Table 3).

Furthermore, when examining each stage of CKD, there was a significant increase in all-cause in-hospital mortality odds in a dose-response trend with CKD stage 3, aOR 1.22 (95% CI: 1.07–1.39, *p* = 0.004); CKD stage 4, aOR 1.43, (95% CI: 1.33–1.66, *p* < 0.001); CKD stage 5, aOR 1.61 (95% CI: 1.02–2.54, *p* = 0.04) (Supplementary Table S6).

#### 3.4.2. Acute respiratory failure

The overall incidence of acute respiratory failure among hospitalized adult patients with COVID-19 was 56.5%. The rate was 56.4% in the kidney disease-free group, 56.1% in the advanced CKD group, 54.8% in the ESKD group, and 46.4% in the KT group (Table 2).

The factor of chronic liver disease was excluded from subsequent multivariable regression models due to its *p*-values being greater than 0.2 in univariate regression. The adjusted logistic regression analysis revealed significantly decreased odds for acute respiratory failure in the advanced CKD, ESKD and KT groups when compared to the kidney disease-free group (aOR 0.92, 95% CI: 0.88–0.96, *p* < 0.001 for advanced CKD; aOR 0.94, 95% CI: 0.89–0.99, *p* = 0.014 for ESKD, aOR: 0.72, 95% CI: 0.62–0.83, *p* < 0.001 for KT) (Table 3).

#### 3.4.3. Acute respiratory distress syndrome

The overall incidence of ARDS among hospitalized COVID-19 adult patients was 5.3%. In the kidney disease-free group, the incidence rate was 5.1%, while it was 6.3% in the advanced CKD group, 6.3% in the ESKD group, and 6.2% in the KT group (Table 2).

The factors of household income, hypertension, and chronic lung disease were excluded from subsequent multivariable regression models due to their *p*-values being greater than 0.2 in univariate regression. The adjusted logistic regression analysis demonstrated a 17% increase in the odds of ARDS in the advanced CKD group compared to the kidney disease-free group (aOR 1.17, 95% CI: 1.07–1.28, *p* < 0.001). No significant difference was found between the ESKD/KT group and the kidney disease-free group (aOR 1.07, 95% CI: 0.96–1.20, *p* = 0.233 for ESKD; aOR 1.19, 95% CI: 0.89–1.58, *p* = 0.235 for KT; Table 3).

#### 3.4.4. Mechanical ventilation

Mechanical ventilation was required for 8.6% of all hospitalized COVID-19 adult patients. The mechanical ventilation rate was higher among patients with advanced CKD, ESKD, and KT at 11.5%, 12.8%, and 8.0% respectively, compared to 7.8% in the kidney disease-free group (Table 2).

The factors of hospital region and bed size were excluded from subsequent multivariable regression models due to their *p*-values being greater than 0.2 in univariable regression. The adjusted logistic regression analysis revealed an 11% and 29% increase in the likelihood of mechanical ventilation in the advanced CKD and ESKD groups compared to the kidney disease-free group (aOR 1.11, 95% CI: 1.04–1.19, *p* = 0.002 for advanced CKD; aOR 1.29, 95% CI: 1.19–1.40, *p* < 0.001 for ESKD). However, there was no statistically significant difference between the KT group and the kidney disease-free group (aOR 1.02, 95% CI: 0.80–1.30, *p* = 0.886) (Table 3).

Notably, CKD stage 4 (aOR 1.19, 95% CI: 1.07–1.32, *p* = 0.001) and CKD stage 5 (aOR 1.56, 95% CI: 1.16–2.08, *p* = 0.003) demonstrated a significant increase in the odds of requiring mechanical ventilation, displaying a dose–response trend (Supplementary Table S6).

#### 3.4.5. Vasopressor requirements

Vasopressor medications were required for 1.8% of all hospitalized adult patients with COVID-19. The use of vasopressors was higher among patients with advanced CKD, ESKD, and KT groups, at 2.6%, 4.0%, and 1.8% respectively, compared to 1.6% among kidney disease-free patients (Table 2).

Chronic lung disease was excluded from subsequent multivariable regression models due to its *p*-values being greater than 0.2 in univariable regression. The adjusted logistic regression analysis indicated a 31% and 96% heightened likelihood of vasopressor use in the advanced CKD and ESKD group respectively, compared to the kidney disease-free group (aOR 1.31, 95% CI: 1.15–1.49, *p* < 0.001 for advanced CKD; aOR 1.96, 95% CI: 1.68–2.28, *p* < 0.001 for advanced ESKD). However, there was no statistically significant difference in vasopressor requirements observed between the KT group and the kidney disease-free group (aOR 1.01, 95% CI: 0.62–1.66, *p* = 0.957 for KT) (Table 3).

CKD stage 3 (aOR 1.27, 95% CI: 1.10–1.48, *p* = 0.002), CKD stage 4 (aOR 1.29, 95% CI: 1.02–1.64, *p* = 0.032) and CKD stage 5 (aOR 2.18, 95% CI: 1.27–3.74, *p* = 0.005) all exhibited a significant increase in the odds of vasopressor requirement, demonstrating a dose–response trend (Supplementary Table S6).

### 3.5. Resource utilization

#### 3.5.1. Length of hospital stay

The overall mean LOS for hospitalized adult patients with COVID-19 was 7.5 days. The LOS was 7.2 days for kidney disease-free patients, 9.0 days for those with advanced CKD, 10.3 days for those with ESKD, and 7.0 days for KT patients (Table 2). All variables had a *p*-value less than 0.2 and were thus included in the subsequent multivariable regression models. The adjusted linear regression analysis indicated that patients with advanced CKD or ESKD had significantly longer hospital stays compared to kidney disease-free patients [adjusted mean difference (aMD): 0.88, 95% CI: 0.70–1.06, *p* < 0.001 for advanced CKD; aMD: 2.31, 95% CI: 2.01–2.61, *p* < 0.001 for ESKD]. However, there were no significant differences in LOS between the KT group and the kidney disease-free group (aMD −0.18, 95% CI: −0.69 to 0.33, *p* = 0.492 for KT) (Table 3).

#### 3.5.2. Total hospitalization charges

The mean total hospitalization charge for hospitalized adult patients with COVID-19 was $79,101. The charges were $76,511 for kidney disease-free patients, $87,470 for those with advanced CKD, $127,376 for those with ESKD, and $81,760 for KT recipients (Table 2). Solid tumors without metastasis and metastasis were excluded from subsequent multivariate regression models due to their *p*-values being greater than 0.2 in univariable regression. Adjusted linear regression analysis revealed a significant increase in hospitalization charges in the advanced CKD and ESKD group compared to the kidney disease-free group (aMD: $4,849, 95% CI: $1,960–$7,738, *p* = 0.001 for advanced CKD, aMD: $37,268, 95% CI: $31,938–$42,598, *p* < 0.001 for ESKD). However, no statistically significant difference was observed between the KT group and the kidney disease-free group (aMD: $2,952, 95% CI: -$5,862 to $11,767, *p* = 0.511 for KT) (Table 3).

### 3.6. Sensitivity analysis of primary and secondary outcomes

We conducted a sensitivity analysis, incorporating confounders that met the following criteria: they have an association with kidney disease, they are identified as risk factors for in-hospital mortality and other adverse outcomes, and they are neither resultant outcomes of kidney disease nor factors within the causal pathway connecting kidney disease to these outcomes. The confounders incorporated into our analysis encompassed age, race, obesity, hypertension, diabetes, coronary artery disease, chronic heart failure, chronic lung disease, and chronic liver disease. The adjusted odds ratio (aOR) derived from the multivariable analysis is detailed in Supplementary Tables S7–S9. Our sensitivity analysis yielded outcomes consistent with our primary analysis, affirming the initial conclusions.

## 4. Discussion

In this study, utilizing the largest inpatient database in the United States, we provide an in-depth overview of the characteristics of patients hospitalized with COVID-19 during its 2020 peak. Encompassing over a million patients, this represents the most exhaustive comparative analysis to date. The study primarily focuses on comparing in-hospital mortality and clinical outcomes among patients with CKD, ESKD, and KT against a control cohort without any underlying kidney conditions. Our study yielded four significant findings. First, comorbidities and multimorbidity were prevalent among patients with CKD, ESKD, and KT, and were associated with increased in-hospital mortality. Furthermore, we described, for the first time to our knowledge, the distribution of comorbidities across the entire spectrum of CKD, ESKD and KT using national U.S. data. Second, the highest in-hospital mortality rate was observed in the advanced CKD group (20.6%), followed by the ESKD group (19.4%) and the KT group (12.4%), all of which were higher than the kidney disease-free group (9.3%). Third, CKD stages 3–5, ESKD, and KT were identified as independent risk factors of mortality, with CKD stage 5 having the highest adjusted odds ratio (aOR) (2.66) for mortality among all variables investigated, surpassing even ESKD. Lastly, a dose-response relationship was observed, wherein the odds of mortality, septic shock, requirement for mechanical ventilation, and vasopressors increased with the increase of CKD stages from 3 to 5.

Our study underscores the potential for severe clinical outcomes due to COVID-19, with an overall 11.1% in-hospital all-cause mortality rate among patients admitted for COVID-19 in the US during the pre-vaccine era, 8.6% receiving invasive mechanical ventilation, and 1.8% requiring vasopressors. The mean hospital LOS was 7.5 days, with an average hospital charge of 79,101 US dollars. The mortality rate estimated in our study is slightly lower than the previously reported 13%–25% in published US studies (18–20). For example, a comprehensive retrospective analysis by Nguyen et al. (18), which utilized data from the Vizient clinical database (an administrative database), encompassing 192,550 COVID-19 hospitalizations in the United States spanning from March to August 2020, reported an in-hospital mortality rate of 13.6%. Several potential explanations could account for this discrepancy. As the pandemic evolved, there were notable advancements in therapeutic interventions, likely leading to improved patient outcomes. Supporting this theory, the study by Nguyen et al. (18) showed a remarkable monthly reduction in mortality during their 6 months period, with the highest mortality in March at 22.1% and dropping to 6.5% by August. Additionally, the varying virulence of different SARS-CoV-2 strains should be considered. Initial strains might have been more virulent, contributing to higher mortality, while subsequent strains could have been less lethal.

In our study, we found that advanced age, male gender, obesity, diabetes, chronic heart failure, chronic lung disease, chronic liver disease, and malignancy were associated with an increased risk of mortality, which aligns with previous reports (2–4). The compromised cardiopulmonary health and impaired immune response associated with these conditions likely contribute to the heightened risk observed. Despite being the most common comorbidity, hypertension was not linked to an increased mortality risk in our study, consistent with previous studies (3). Our study identified chronic liver disease and CKD stage 5 as the most significant risk factors for mortality, each with an aOR greater than 2.5. This observation is supported by a meta-analysis that revealed a comparable pooled odds ratio of 2.35 for mortality associated with chronic liver disease among COVID-19 patients (21). Moreover, to the best of our knowledge, we are the first to describe the distribution of comorbidities across the entire spectrum of CKD, ESKD, and KT using national U.S. data. For example, 57.7% of individuals with CKD stage 3 have diabetes and 90.3% of advanced CKD patients had a CCI equal to or greater than three. These insights are vital for strategizing treatment and prevention services in individuals with kidney disease, both during and after the pandemic.

The study also highlighted significant disparities influenced by race/ethnicity and socioeconomic status in COVID-19-related hospitalizations and fatalities. For instance, Native Americans had the highest mortality associated with COVID-19 among all ethnic groups, consistent with existing literature (22, 23), emphasizing the need for targeted interventions and support for Native American populations. Although some studies suggest higher mortality rates among Black individuals (24), another national study by Pal et al. (25) found similar mortality risks for Black and White individuals, aligning with our observations. This discrepancy could result from variations in the timing of data collection across different studies (25). The correlation between larger urban hospitals and increased mortality could be due to a higher burden of severely ill patients in larger hospitals, potentially reflecting the higher volume and complexity of cases treated in urban centers and revealing a deficiency in the US healthcare system’s capacity to adequately serve affected populations during the pandemic. The association between low income and heightened mortality underscores the impact of socioeconomic status on health outcomes, highlighting the need for a multidimensional approach to address the underlying inequities that exacerbate health disparities during crises like the COVID-19 pandemic. Policymakers and healthcare professionals must collaboratively address the factors contributing to these disparities comprehensively.

The COVID-19 pandemic has disproportionately impacted patients with kidney disease. Earlier studies have identified CKD as an independent risk factor for SARS-COV-2 infection, increased mortality, and adverse COVID-19 outcomes (7, 8, 26). According to the 2022 Annual Data Report from the United States Renal Data System, there was a significant increase in mortality rates among individuals with CKD across all stages. The rate increased from 91.7 per 1,000 person-years in 2019 to 100.6 per 1,000 person-years in 2020. Notably, this rise was more pronounced in individuals with CKD stages 4 and 5, where the mortality rate surged from 161.8 per 1,000 to 180.5 per 1,000. Additionally, a systematic review reported a pooled odds ratio (OR) of 1.77 for mortality risk among patients with CKD and COVID-19 (7). However, comprehensive evaluations of COVID-19 outcomes across different CKD stages, particularly considering major comorbidities, are still lacking. Our analysis addressed this gap by considering demographic and socioeconomic factors, as well as comorbid conditions known to be prevalent in individuals with CKD or increased COVID-19 severity and mortality. We analyzed COVID-19-related mortality and adverse outcomes stratified across the entire spectrum of CKD stages. We found that the group with advanced CKD had the highest mortality rate (20.6%), surpassing the ESKD (19.4%) and KT (12.4%) cohorts, a finding aligned with smaller studies and a large nationwide Turkish analysis (27). Further investigation attributed this increased mortality primarily to CKD stages 4 and 5, which displayed even higher mortality rates of 24.1% and 26.6%, respectively. We also found that hospitalized COVID-19 patients with advanced CKD were, on average, nearly 10 years older than those without kidney disease and had a significantly higher burden of comorbidities. However, after adjustment, advanced CKD demonstrated a 52% increase in the odds of in-hospital mortality, consistent with the literature (7), suggesting that advanced CKD is an independent risk factor for mortality. Interestingly, our analysis indicated a dose–response relationship between mortality odds and advancing CKD stages, with aORs of 1.34, 1.8, and 2.66 for CKD stages 3–5, respectively. Williamson et al. reported that among individuals diagnosed with COVID-19 in the United Kingdom, those with CKD stage 3 had a 33% increased risk of COVID-19-related death compared to those with normal kidney function or mild CKD (stages 1 and 2). Meanwhile, the risk of death for individuals with CKD stages 4 and 5 was 2.5 times higher, a finding that is comparable to our results (3). Regarding adverse outcomes, after adjustments, patients with advanced CKD still showed heightened susceptibility to septic shock, and ARDS, and a higher likelihood of requiring mechanical ventilation and vasopressors. Additionally, we observed a dose-response relationship, where the odds of mortality, septic shock, requirement for mechanical ventilation, and vasopressors increased as CKD stages increased from 3 to 5. These results suggest that CKD stages 3–5 are independent risk factors for COVID-19-related mortality and adverse outcomes. This increased risk of adverse COVID-19 outcomes and mortality among individuals with CKD may partly reflect impaired immunity. Uremia is associated with chronic inflammation and impaired natural and adaptive immunity, leading to increased production and decreased clearance of pro-inflammatory cytokines, which, in turn, contributes to the elevated mortality observed in these patients (28, 29).

Our analysis further indicated that patients with ESKD hospitalized due to COVID-19 exhibited a significantly elevated in-hospital all-cause mortality rate of 19.4%. ESKD was pinpointed as an independent risk factor for mortality (aOR 1.97), septic shock (aOR 2.12), mechanical ventilation (aOR 1.29), and the need for vasopressors (aOR 1.96), and correlated with an extended length of stay by an average of two additional days. Although initial data from Wuhan, China, suggested a modest impact of COVID-19 on dialysis patients, with a mortality rate of 4.7% and predominantly mild clinical symptoms without progression to severe pneumonia (30), subsequent case series from Italy in March 2020 indicated a significantly augmented mortality risk in this demographic (31, 32). Subsequent studies corroborated COVID-19 mortality rates in ESKD patients ranging from 15% to 30% (19, 33–35), which is in line with our findings. For instance, Ghonimi et al. (33) reported a 15% COVID-19-related mortality rate in a dialysis cohort, whereas a larger retrospective study utilizing Centers for Medicare & Medicaid Services (CMS) data noted a post-COVID-19 diagnosis mortality rate of 26.0% among medicare patients undergoing dialysis in 2020, markedly higher than in dialysis patients without COVID-19 (16.9%) (35). Our results, indicating an approximately two-fold higher adjusted in-hospital mortality in the ESKD group compared to the group without kidney disease, are in accordance with the literature (27).

Our study included 4,450 hospitalized COVID-19 patients who were KT recipients, with an average age of 59 years. We observed an in-hospital all-cause mortality rate of 12.4%, which is higher than the 9.3% rate observed in the cohort without kidney disease and the 11.1% rate observed in the general population. After adjusting for confounding variables, the mortality odds for KT recipients were 1.69 times higher than for patients without kidney disease, and the odds of developing septic shock were 1.42 times higher. This is consistent with Fisher’s et al. (36) findings, which indicated a higher risk of death among transplant recipients compared to non-transplanted patients [adjusted odds ratio (aOR) 1.93] in the United States. Other studies have reported mortality rates significantly higher than our analysis, showcasing a wide range of COVID-19 mortality rates among KT patients. For example, the TANGO International Transplant Consortium reported a 32% mortality rate over a median follow-up of 52 days for 144 hospitalized KT patients with COVID-19 (13). A meta-analysis by Kremer et al. (14) involving 5,559 COVID-19 KT patients, of whom 86% were hospitalized, estimated a 23% mortality rate. In contrast, a nationwide Turkish analysis reported an in-hospital mortality rate of 11.1% (27). This variability could result from differences in sample sizes, reporting methodologies (e.g., all-cause mortality, 30 days mortality, COVID-19-related mortality, inpatient versus outpatient mortality), or an underestimation of SARS-CoV-2 incidence early in the pandemic due to limited testing, potentially leading to an overestimation of mortality. For instance, a single-center study found that COVID-19-related mortality decreased from 32% to 15% when including cases identified via serology testing (37). The discrepancy between our study and earlier ones could be due to our larger sample size or the lack of post-discharge follow-up data, precluding late morbidity and mortality analysis. It is also essential to note that differences in transplant duration, donor sources, CKD stages post-transplant, and COVID-19 treatment approaches might have affected the outcomes and served as confounders. For example, some studies have shown that death risk varies with the time since transplantation (14, 38); however, these key pieces of information were not available in the NIS database. Additionally, transplant recipients may have different clinical presentation, hospitalization, and treatment thresholds than the general population (15). KT patients, irrespective of symptoms, are more likely to undergo COVID-19 testing and might have a lower threshold for hospitalization than the general population. For example, research has shown that transplant recipients had lower WHO severity scores and peak CRP levels upon COVID-19 admission (39, 40). Moreover, a higher percentage of transplant recipients did not require oxygen upon COVID-19 admission (39, 40), which is consistent with our findings (aOR 0.72 for acute respiratory failure in the KT group). This might suggest that a substantial number of transplant recipients were hospitalized primarily due to gastrointestinal symptoms related to COVID-19 and/or graft dysfunction, both associated with a more favorable prognosis (41, 42). Research also indicated that KT patients were significantly more likely to receive specific COVID-19 treatments like tocilizumab, which has been proven to reduce mortality in severe COVID-19 cases (39, 40). Additionally, many transplant recipients received prednisone as part of their immunosuppression regimen, and corticosteroids are currently a standard treatment for hospitalized COVID-19 patients (43). These factors may have a positive impact on the mortality rate among KT recipients. Therefore, the reported mortality rate among KT patients should be interpreted with caution due to significant risks of collider bias and residual confounding factors.

Despite the strengths of our study, such as its large, nationwide sample size and comprehensive scope, there are several notable limitations. First, our data were extracted from an administrative database, which does not contain detailed clinical information. As a result, important information such as the cause of kidney disease, stages of CKD in the kidney allograft, the age of the transplant, and medication usage were unavailable. Additionally, the identification of clinical conditions and procedures depended on the accuracy of the diagnoses and procedure codes reported by the hospitals, making the database vulnerable to inaccuracies or missing codes. Second, due to the retrospective observational nature of this study, exposures could not be randomized. Although we employed regression analysis to adjust for confounders, the possibility of residual confounding remains. Lastly, while the findings of this study are representative of the clinical outcomes of adult patients hospitalized for COVID-19 in the United States, outcomes may differ in other settings or regions around the world.

## 5. Conclusion

In conclusion, our nationwide study highlights a greater burden of multimorbidity in COVID-19 patients hospitalized with advanced CKD, ESKD, and KT, compared to those without pre-existing kidney disease. We meticulously detailed the distribution of comorbidities across all stages of CKD, ESKD, and KT, thereby providing essential insights necessary for strategizing treatment and preventive services for individuals with kidney disease, during and beyond the pandemic. Moreover, our findings indicate higher in-hospital mortality rates in patients with advanced CKD, ESKD, and KT compared to those without kidney disease. Stages 3 to 5 CKD, ESKD, and KT emerged as independent risk factors of mortality, illustrating a dose-response relationship between increasing CKD stages (from 3 to 5) and the odds of mortality and adverse outcomes. This study emphasizes the necessity for targeted interventions to mitigate the risk of adverse outcomes in the vulnerable population of patients with CKD, ESKD, and KT. It is imperative that further research be conducted to understand the long-term effects of COVID-19 on these populations and to develop strategies to optimize their care and outcomes.

## Data availability statement

Publicly available datasets were analyzed in this study. This data can be found at: https://hcup-us.ahrq.gov/news/announcements/nis2020.jsp.

## Ethics statement

Ethical approval was not required for the studies involving humans because approval from an Institutional Review Board was not required for this study due to its retrospective nature and the use of de-identified data. The study was conducted in strict adherence to the data-use agreements of the NIS-HCUP. The studies were conducted in accordance with the local legislation and institutional requirements. Written informed consent for participation was not required from the participants or the participants’ legal guardians/next of kin in accordance with the national legislation and institutional requirements because approval from an Institutional Review Board was not required for this study for the aforementioned reason. The study was conducted in strict adherence to the data-use agreements of the NIS-HCUP.

## Author contributions

MH conceived the study, accessed, verified the data, led the data analysis with support from AG, and led the writing of the paper. YW, SL, and AG commented on the paper, oversaw the analysis, and edited the final manuscript. All authors contributed to the article and approved the submitted version.

## Funding

Publication of this article was funded in part by the Temple University Libraries Open Access Publishing Fund. We sincerely appreciate the support.

## Abbreviations

COVID-19: Coronavirus disease 2019
SARS-CoV-2: Severe acute respiratory syndrome coronavirus-2
CKD: Chronic kidney disease
ESKD: End stage kidney disease
KT: Kidney transplant
HCUP: Healthcare Cost and Utilization Project
NIS: Nationwide Inpatient Sample
ICD-10-CM: International Classification of Diseases, 10th revision, Clinical Modification
ARDS: Acute respiratory distress syndrome
LOS: Length of hospital stay
CCI: Charlson’s comorbidity index
CI: Confidence interval
aOR: Adjusted odds ratio
aMD: Adjusted mean difference
