# Supplementary material for: Nationwide in-hospital mortality and morbidity analysis of COVID-19 in advanced chronic kidney disease, dialysis and kidney transplant recipients
Source: Front Med (Lausanne). 2023 Nov 2;10:1250631. doi: 10.3389/fmed.2023.1250631 (PMC10652751; doi:10.3389/fmed.2023.1250631)
Supplement: Supplementary file 1 [file Data_Sheet_1.docx]

Supplementary Material

# Supplementary Table S1

ICD‐10‐CM codes for diseases and procedures used in the study.

| **Diagnosis** | | **ICD-10 code** |
| --- | --- | --- |
| Covid 19 | | U071, U00, U49, U50, U85, J1282 |
| Chronic kidney disease (CKD)  Stage 1 | | N181 |
| CKD Stage 2 | | N182 |
| CKD Stage 3 | | N183 |
| CKD Stage 4 | | N184 |
| CKD Stage 5 | | N185 |
| Advanced CKD | | N183, N184, N185 |
| ESKD | | N186, Z992 |
| Kidney transplant | | Z940 |
| Renal disease | | I120, I131, N032 - N037, N052 - N057, N18X, N19X, N250, Z490 - Z492, Z940, Z992 |
| Congestive heart failure | | I099, I110, I130, I132, I255, I420, I425 - I429, I43X, I50X, P290 |
| Coronary artery disease | | I25 |
| Chronic pulmonary disease | | I278, I279, J40X - J47X, J60X - J67X, J684, J701, J703 |
| Diabetes mellitus: | Diabetes, uncomplicated | E100, E101, E109, E110, E111, E119, E120, E121, E129, E130, E131, E139, E140, E141, E149 |
|  | Diabetes, complicated | E102 - E108, E112 - E118, E122 - E128, E132 - E138, E142 - E148 |
| Hypertension | | I10X, I11X, I12X, I13X, I15X |
| Liver disease | | B18X, I85X, I864, I982, K70X, K711, K713 - K715, K717, K72X - K74X, K760, K762 - K769, Z944 |
| Obesity | | E66X |
| Solid tumor | | C00X - C26X, C30X - C34X, C37X - C41X, C43X, C45X - C58X, C60X - C76X, C97X |
| Metastasis Cancer | | C77X - C80X |
| Alcohol abuse | | F10, E52, G621, I426, K292, K700, K703, K709, T51X, Z502, Z714, Z721 |
| Smoking | | F172, Z720, Z87891 |
| Drug abuse | | F11X - F16X, F18X, F19X, Z715, Z722 |
| Septic shock | | R6521 |
| Acute respiratory failure | | J9600, J9601, J9602, J9620, J9621, J9622 |
| Acute Respiratory Distress Syndrome (ARDS) | | J80 |
| Mechanical ventilation | | 5A1945Z,5A1955Z,5A1935Z,5A09357,5A09457,5A09557 |

# Supplementary Table S2

Baseline characteristics of adult patients hospitalized for COVID-19

|  |  | **Total N= 1,018,915** | **Kidney disease-free N= 813,110**  **(79.8%)** | **Advanced CKD N= 58,780 (5.8%)** | **ESKD N= 38,370 (3.8%)** | **KT N= 4450 (0.4%)** | **P- value** |
| --- | --- | --- | --- | --- | --- | --- | --- |
| **Age (year)** |  | 64.7 ± 0.1 | 62.8 ± 0.1 | 74.3 ± 0.1 | 64.0 ± 0.2 | 59.1 ± 0.4 | <0.001 |
| **Female Sex n (%)** |  | 480,540 (47.2%) | 390,900 (48.1%) | 27,865 (46.6%) | 17,120 (43.6%) | 1,580 (35.5%) | <0.001 |
| **Race n (%)** |  |  |  |  |  |  | <0.001 |
|  | **White** | 518,470 (52.4%) | 411,590 (52.2%) | 31,090 (53.5%) | 11,315 (29.6%) | 1,695 (39.2%) |  |
|  | **Black** | 183,825 (18.6%) | 134,005 (17.0%) | 16,075 (27.2%) | 13,190 (34.4%) | 1,130 (26.1%) |  |
|  | **Hispanic** | 204,095 (20.6%) | 174,425 (22.1%) | 7,495 (12.6%) | 9,880 (25.8%) | 1,135 (26.2%) |  |
|  | **Asian or Pacific Islander** | 32,370 (3.3%) | 26,480 (3.4%) | 1,725 (2.9%) | 1,530 (4.0%) | 145 (3.4%) |  |
|  | **Native American** | 10,160 (1.0%) | 8,230 (1.0%) | 355 (0.6%) | 850 (2.3%) | 70 (1.6%) |  |
|  | **Others** | 40,210 (4.1%) | 34,090 (4.3%) | 1,815 (3.1%) | 1,535 (4.0%) | 150 (3.5%) |  |
| **Median household income for patient’s zip code n (%)** |  |  |  |  |  |  | <0.001 |
|  | **$ 1–$ 49,999** | 339,825 (33.9%) | 267,995 (33.5%) | 21,080 (35.5%) | 16,245 (42.4%) | 1,550 (35.2%) |  |
|  | **$ 50,000–$ 64,999** | 276,400 (27.6%) | 220,260 (27.5%) | 15,690 (26.5%) | 10,235 (26.5%) | 1200 (27.3%) |  |
|  | **$ 65,000–$ 85,999** | 222,140 (22.2%) | 178,000 (22.3%) | 12,680 (21.3%) | 7,545 (19.5%) | 910 (20.7%) |  |
|  | **$ 86,000 or more** | 164,555 (16.4%) | 133,830 (16.7%) | 9,840 (16.7%) | 4,520 (11.7%) | 740 (16.8%) |  |
| **Insurance status n (%)** |  |  |  |  |  |  | <0.001 |
|  | **Medicare** | 531,325 (54.9%) | 377,985 (49.3%) | 45,410 (77.9%) | 28,430 (74.2%) | 2,935 (67.9%) |  |
|  | **Medicaid** | 119,350 (12.3%) | 103,300 (13.5%) | 4,330 (7.4%) | 4,795 (12.6%) | 400 (9.3%) |  |
|  | **Private including HMO** | 281,625 (29.1%) | 253,565 (33.1%) | 8,000 (13.5%) | 4,560 (11.8%) | 940 (21.7%) |  |
|  | **Self- Pay** | 34,875 (3.6%) | 32,375 (4.2%) | 750 (1.3%) | 505 (1.3%) | 50 (1.2%) |  |
| **Comorbidities n (%)** |  |  |  |  |  |  |  |
|  | **Congestive heart failure** | 171,070 (16.8%) | 93,150 (11.5%) | 24,810 (41.4%) | 17,275 (44.2%) | 960 (21.6%) | <0.001 |
|  | **Coronary artery disease** | 187,390 (18.4%) | 117,755 (14.5%) | 20,805 (34.7%) | 13,270 (34.1%) | 1,030 (23.2%) | <0.001 |
|  | **Chronic pulmonary disease** | 239,610 (23.5%) | 184,315 (22.7%) | 16,645 (28.0%) | 8,445 (21.6%) | 495 (11.1%) | <0.001 |
|  | **Diabetes** | 415,645 (40.8%) | 293,805 (36.1%) | 35,975 (59.6%) | 27,980 (71.6%) | 2,490 (56.0%) | <0.001 |
|  | **Hypertension** | 690,155 (67.7%) | 502,070 (61.8%) | 55,565 (92.4%) | 37,375 (95.2%) | 3,855 (86.6%) | <0.001 |
|  | **Solid tumor without metastasis** | 20,390 (2.0%) | 15,375 (1.9%) | 1,425 (2.4%) | 650 (1.7%) | 65 (1.5%) | <0.001 |
|  | **Metastatic cancer** | 8,010 (0.8%) | 6,210 (0.8%) | 510 (0.9%) | 245 (0.6%) | 30 (0.7%) | 0.006 |
|  | **Chronic Liver disease** | 46,480 (4.6%) | 36,335 (4.5%) | 2,745 (4.6%) | 2,535 (6.5%) | 265 (6.0%) | <0.001 |
|  | **Obesity** | 280,870 (27.6%) | 230,195 (28.3%) | 14,760 (24.6%) | 8,770 (22.5%) | 810 (18.2%) | <0.001 |
|  | **Alcohol use** | 19,325 (1.9%) | 16,305 (2.0%) | 830 (1.4%) | 495 (1.3%) | 35 (0.8%) | <0.001 |
|  | **Smoking** | 223,720 (22.0%) | 170,990 (21.0%) | 15,055 (25.2%) | 8,190 (20.8%) | 950 (21.4%) | <0.001 |
|  | **Drug use** | 18,730 (1.8%) | 15,550 (1.9%) | 935 (1.5%) | 705 (1.8%) | 45 (1.0%) | <0.001 |
| **Charlson Comorbidity Index (CCI) n (%)** |  |  |  |  |  |  | <0.001 |
|  | **0** | 282,400 (27.7%) | 282,400 (34.7%) | 0 (0%) | 0 (0%) | 0 (0%) |  |
|  | **1** | 283,960 (27.9%) | 283,960 (34.9%) | 0 (0%) | 0 (0%) | 0 (0%) |  |
|  | **2** | 166,160 (16.3%) | 142,915 (17.6%) | 5,905 (9.7%) | 3,590 (9.0%) | 1,105 (24.8%) |  |
|  | **>=3** | 286,395 (28.1%) | 103,835 (12.8%) | 54,215 (90.3%) | 35,705 (91.0%) | 3,345 (75.2%) |  |
| **Hospital bed size n (%)** |  |  |  |  |  |  | <0.001 |
|  | **Small** | 256,360 (25.2%) | 206,495 (25.4%) | 14,475 (24.1%) | 8,020 (20.4%) | 825 (18.5%) |  |
|  | **Medium** | 295,700 (29.0%) | 237,465 (29.2%) | 17,225 (28.6%) | 11,485 (29.2%) | 1,165 (26.2%) |  |
|  | **Large** | 466,855 (45.8%) | 369,150 (45.4%) | 28,420 (47.3%) | 19,790 (50.4%) | 2,460 (55.3%) |  |
| **Hospital Location/teaching status n (%)** |  |  |  |  |  |  | <0.001 |
|  | **Rural** | 110,395 (10.8%) | 89,060 (11.0%) | 5,500 (9.2%) | 2,300 (5.8%) | 330 (7.4%) |  |
|  | **Urban nonteaching** | 196,855 (19.3%) | 159,240 (19.6%) | 10,695 (17.8%) | 7,025 (18.0%) | 560 (12.6%) |  |
|  | **Urban teaching** | 711,665 (69.9%) | 564,810 (69.5%) | 43,925 (73.0%) | 29,970 (76.2%) | 3,560 (80.0%) |  |
| **Hospital Region n (%)** |  |  |  |  |  |  | <0.001 |
|  | **Northeast** | 183,195 (18.0%) | 146,355 (18.0%) | 11,460 (19.0%) | 7060 (18.0%) | 890 (20.0%) |  |
|  | **Midwest** | 236,315 (23.2%) | 180,185 (22.2%) | 15,050 (24.9%) | 8,180 (20.9%) | 1,120 (25.2%) |  |
|  | **South** | 422,431 (41.5%) | 341,346 (42.0%) | 25,685 (42.9%) | 16,410 (41.7%) | 1,645 (37.0%) |  |
|  | **West** | 176,974 (17.4%) | 145,225 (17.9%) | 7,925 (13.2%) | 7,645 (19.5%) | 795 (17.9%) |  |

*: Regions of hospital in NIS: please refer to Supplementary Table S3

# Supplementary Table S3

Regions of hospital in NIS

| Region | States |
| --- | --- |
| Northeast | New England, Maine, New Hampshire, Vermont, Massachusetts, Rhode Island, Connecticut, New York, Pennsylvania, New Jersey |
| Midwest | Wisconsin, Michigan, Illinois, Indiana, Ohio, Missouri, North Dakota, South Dakota, Nebraska, Kansas, Minnesota, Iowa |
| South | Delaware, Maryland, District of Columbia, Virginia, West Virginia, North Carolina, South Carolina, Georgia, Florida, Kentucky, Tennessee, Mississippi, Alabama, Oklahoma, Texas, Arkansas, Louisiana |
| West | Idaho, Montana, Wyoming, Nevada, Utah, Colorado, Arizona, New Mexico, Alaska, Washington, Oregon, California, Hawaii |

# Supplementary Table S4

Baseline characteristics of adult patients hospitalized for COVID-19 stratified by CKD stages.

|  |  | **Kidney disease-free N= 813,110 (79.8%)** | **CKD1**  **N= 955 (0.1%)** | **CKD2**  **N= 10,875 (1.1%)** | **CKD3**  **N= 37,525 (3.7%)** | **CKD4**  **N= 19,200 (1.9%)** | **CKD5**  **N= 2,055 (0.2%)** | **ESKD**  **N= 38,370 (3.8%)** | **P**  **value** |
| --- | --- | --- | --- | --- | --- | --- | --- | --- | --- |
| **Age (year)** |  | 62.8 ± 0.1 | 67.3 ± 1.0 | 70.5 ± 0.3 | 74.1 ± 0.2 | 75.0 ± 0.2 | 69.5 ± 0.7 | 64.0 ± 0.2 | <0.001 |
| **Female Sex n (%)** |  | 390,900 (48.1%) | 430 (44.5%) | 4,005 (36.6%) | 16,960 (44.5%) | 9,840 (50.4%) | 1,135 (50.4%) | 17,120 (43.6%) | <0.001 |
| **Race n (%)** |  |  |  |  |  |  |  |  | <0.001 |
|  | **White** | 411,590 (52.2%) | 490 (51.9%) | 5,780 (54.1%) | 19,465 (52.6%) | 10,880 (56.9%) | 825 (39.4%) | 11,315 (29.6%) |  |
|  | **Black** | 134,005 (17.0%) | 210 (22.2%) | 2,960 (27.5%) | 10,520 (28.1%) | 4,805 (24.7%) | 790 (34.9%) | 13,190 (34.4%) |  |
|  | **Hispanic** | 174,425 (22.1%) | 145 (15.7%) | 1,300 (12.2%) | 4,805 (12.7%) | 2,345 (12.1%) | 365 (15.7%) | 9,880 (25.8%) |  |
|  | **Asian or Pacific Islander** | 26,480 (3.4%) | 45 (4.9%) | 310 (2.9%) | 1,105 (3.0%) | 540 (2.8%) | 80 (3.7%) | 1,530 (4.0%) |  |
|  | **Native American** | 8,230 (1.0%) | 5 (0.5%) | 65 (0.6%) | 200 (0.5%) | 120 (0.6%) | 40 (1.5%) | 850 (2.3%) |  |
|  | **Others** | 34,090 (4.3%) | 45 (4.9%) | 290 (2.7%) | 1,155 (3.1%) | 570 (3.0%) | 95 (4.7%) | 1,535 (4.0%) |  |
| **Median household income for patient’s zip code n (%)** |  |  |  |  |  |  |  |  | <0.001 |
|  | **$ 1–$ 49,999** | 267,995 (33.5%) | 310 (32.1%) | 3,825 (35.2%) | 13,125 (34.7%) | 7,075 (36.2%) | 945 (42.1%) | 16,245 (42.4%) |  |
|  | **$ 50,000–$ 64,999** | 220,260 (27.5%) | 195 (20.9%) | 3,035 (28.0%) | 9,970 (26.6%) | 5,195 (26.6%) | 550 (25.4%) | 10,235 (26.5%) |  |
|  | **$ 65,000–$ 85,999** | 178,000 (22.3%) | 255 (27.3%) | 2,410 (22.3%) | 7,965 (21.1%) | 4,355 (22.1%) | 410 (18.2%) | 7,545 (19.5%) |  |
|  | **$ 86,000 or more** | 133,830 (16.7%) | 190 (19.8%) | 1,610 (14.6%) | 6,595 (17.6%) | 2,940 (15.2%) | 315 (14.3%) | 4,520 (11.7%) |  |
| **Insurance status n (%)** |  |  |  |  |  |  |  |  | <0.001 |
|  | **Medicare** | 377,985 (49.3%) | 590 (63.9%) | 7,510 (70.8%) | 28,560 (76.9%) | 15,480 (80.6%) | 1,475 (69.7%) | 28,430 (74.2%) |  |
|  | **Medicaid** | 103,300 (13.5%) | 120 (13.1%) | 850 (8.0%) | 2,640 (7.0%) | 1,340 (7.0%) | 355 (16.4%) | 4,795 (12.6%) |  |
|  | **Private including HMO** | 253,565 (33.1%) | 200 (20.8%) | 2,085 (19.7%) | 5,460 (14.6%) | 2,265 (11.4%) | 310 (13.6%) | 4,560 (11.8%) |  |
|  | **Self- Pay** | 32,375 (4.2%) | 20 (2.2%) | 155 (1.5%) | 535 (1.5%) | 210 (1.0%) | 5 (0.3%) | 505 (1.3%) |  |
| **Comorbidities n (%)** |  |  |  |  |  |  |  |  |  |
|  | **Congestive heart failure** | 93,150 (11.5%) | 265 (27.8%) | 3205 (29.2%) | 14,325 (37.5%) | 9,625 (49.0%) | 925 (41.6%) | 17,275 (44.2%) | <0.001 |
|  | **Coronary artery disease** | 117,755 (14.5%) | 270 (28.3%) | 3,100 (28.1%) | 12,800 (33.4%) | 7,495 (38.1%) | 580 (25.8%) | 13,270 (34.1%) | <0.001 |
|  | **Chronic pulmonary disease** | 184,315 (22.7%) | 310 (31.4%) | 3,095 (28.4%) | 10,760 (28.4%) | 5,515 (28.2%) | 430 (19.5%) | 8,445 (21.6%) | <0.001 |
|  | **Diabetes** | 293,805 (36.1%) | 600 (62.8%) | 5,805 (52.6%) | 22,160 (57.7%) | 12,505 (63.1%) | 1435 (62.5%) | 27,980 (71.6%) | <0.001 |
|  | **Hypertension** | 502,070 (61.8%) | 815 (84.3%) | 9,640 (87.3%) | 35,480 (92.7%) | 18,075 (91.4%) | 2,155 (95.6%) | 37,375 (95.2%) | <0.001 |
|  | **Solid tumor without metastasis** | 15,375 (1.9%) | 25 (2.6%) | 325 (3.0%) | 925 (2.4%) | 475 (2.4%) | 30 (1.2%) | 650 (1.7%) | <0.001 |
|  | **Metastatic cancer** | 6,210 (0.8%) | 10 (1.1%) | 145 (1.3%) | 370 (1.0%) | 125 (0.6%) | 15 (0.5%) | 245 (0.6%) | 0.004 |
|  | **Chronic Liver disease** | 36,335 (4.5%) | 55 (5.2%) | 485 (4.3%) | 1,750 (4.6%) | 925 (4.7%) | 80 (3.4%) | 2,535 (6.5%) | <0.001 |
|  | **Obesity** | 230,195 (28.3%) | 265 (27.8%) | 3,210 (29.2%) | 9,575 (25.0%) | 4,710 (23.8%) | 510 (23.1%) | 8,770 (22.5%) | <0.001 |
|  | **Alcohol use** | 16,305 (2.0%) | 20 (2.1%) | 240 (2.2%) | 565 (1.5%) | 260 (1.3%) | 15 (0.7%) | 495 (1.3%) | <0.001 |
|  | **Smoking** | 170,990 (21.0%) | 255 (26.7%) | 2970 (27.0%) | 9,965 (26.2%) | 4,680 (23.8%) | 455 (19.5%) | 8,190 (20.8%) | <0.001 |
|  | **Drug use** | 15,550 (1.9%) | 25 (2.6%) | 200 (1.8%) | 635 (1.7%) | 270 (1.3%) | 35 (1.5%) | 705 (1.8%) | <0.001 |
| **Charlson Comorbidity Index (CCI) n (%)** |  |  |  |  |  |  |  |  | <0.001 |
|  | **0** | 282,400 (34.7%) | 0 (0%) | 0 (0%) | 0 (0%) | 0 (0%) | 0 (0%) | 0 (0%) |  |
|  | **1** | 283,960 (34.9%) | 0 (0%) | 0 (0%) | 0 (0%) | 0 (0%) | 0 (0%) | 0 (0%) |  |
|  | **2** | 142,915 (17.6%) | 110 (11.5%) | 1,705 (15.4%) | 4,085 (10.7%) | 1,535 (7.5%) | 285 (12.7%) | 3,590 (9.0%) |  |
|  | **>=3** | 103,835 (12.8%) | 860 (88.5%) | 9,315 (84.6%) | 34,170 (89.3%) | 18,230 (92.5%) | 1,965 (87.4%) | 35,705 (91.0%) |  |
| **Hospital bed size n (%)** |  |  |  |  |  |  |  |  | <0.001 |
|  | **Small** | 206,495 (25.4%) | 240 (24.1%) | 2,660 (24.2%) | 8,965 (23.4%) | 4,965 (25.4%) | 560 (24.1%) | 8,020 (20.4%) |  |
|  | **Medium** | 237,465 (29.2%) | 235 (24.1%) | 2,955 (26.8%) | 11,125 (29.0%) | 5,530 (28.0%) | 630 (28.2%) | 11,485 (29.2%) |  |
|  | **Large** | 369,150 (45.4%) | 495 (51.8%) | 5,405 (49.0%) | 18,165 (47.6%) | 9,270 (46.6%) | 1,060 (47.7%) | 19,790 (50.4%) |  |
| **Hospital Location/teaching status n (%)** |  |  |  |  |  |  |  |  | <0.001 |
|  | **Rural** | 89,060 (11.0%) | 90 (8.9%) | 1,080 (9.9%) | 2,945 (7.8%) | 2,365 (12.0%) | 205 (8.5%) | 2,300 (5.8%) |  |
|  | **Urban nonteaching** | 159,240 (19.6%) | 145 (15.2%) | 1,800 (16.4%) | 6,795 (17.8%) | 3,515 (17.7%) | 410 (19.0%) | 7,025 (18.0%) |  |
|  | **Urban teaching** | 564,810 (69.5%) | 735 (75.9%) | 8,140 (73.8%) | 28,515 (74.4%) | 13,885 (70.3%) | 1,635 (72.5%) | 29,970 (76.2%) |  |
| **Hospital Region n (%)** |  |  |  |  |  |  |  |  | <0.001 |
|  | **Northeast** | 146,355 (18.0%) | 195 (20.4%) | 1,620 (14.7%) | 7,320 (19.2%) | 3,615 (18.3%) | 535 (23.1%) | 7060 (18.0%) |  |
|  | **Midwest** | 180,185 (22.2%) | 215 (22.0%) | 3,395 (30.7%) | 9,085 (23.7%) | 5,560 (28.0%) | 450 (19.2%) | 8,180 (20.9%) |  |
|  | **South** | 341,346 (42.0%) | 355 (37.2%) | 4,300 (39.0%) | 16,840 (44.1%) | 7,980 (40.4%) | 945 (42.6%) | 16,410 (41.7%) |  |
|  | **West** | 145,225 (17.9%) | 205 (20.4%) | 1,705 (15.6%) | 5,010 (13.1%) | 2,610 (13.3%) | 320 (15.1%) | 7,645 (19.5%) |  |

# Supplementary Table S5

Outcomes of adult patients hospitalized for COVID-19 stratified by CKD status.

|  |  | **Kidney disease-free** | **CKD1** | **CKD2** | **CKD3** | **CKD4** | **CKD5** | **ESKD** | **P- value** |
| --- | --- | --- | --- | --- | --- | --- | --- | --- | --- |
| Mortality | All cause in hospital mortality N % | 75,270 (9.3%) | 140 (14.7%) | 1,525 (14.0%) | 6,930 (18.5%) | 4,625 (24.1%) | 545 (26.6%) | 7,445 (19.4%) | <0.001 |
| Morbidity |  |  |  |  |  |  |  |  |  |
|  | Septic shock N % | 24,450 (3.0%) | 50 (5.2%) | 435 (4.0%) | 1,680 (4.5%) | 970 (5.1%) | 125 (6.1%) | 3,075 (8.0%) | <0.001 |
|  | Acute respiratory failure N % | 458,645 (56.4%) | 560 (58.6%) | 6,195 (57.0%) | 20,985 (55.9%) | 10,940 (57.0%) | 1,045 (50.9%) | 21,025 (54.8%) | <0.001 |
|  | ARDS N % | 41,640 (5.1%) | 80 (8.4%) | 630 (5.8%) | 2245 (6.0%) | 1320 (6.9%) | 140 (6.8%) | 2,430 (6.3%) | <0.001 |
|  | Mechanical ventilation N % | 63,640 (7.8%) | 115 (12.0%) | 1,220 (11.2%) | 4,050 (10.8%) | 2385 (12.4%) | 300 (14.6%) | 4,895 (12.8%) | <0.001 |
|  | Vasopressor N % | 13,240 (1.6%) | 20 (2.1%) | 265 (2.4%) | 970 (2.5%) | 515 (2.5%) | 85 (4.1%) | 1,550 (4.0%) | <0.001 |
| Resource Utilization |  |  |  |  |  |  |  |  |  |
|  | Mean LOS (days) | 7.2 ± 0.03 | 9.0 ± 0.77 | 8.2 ± 0.17 | 9.2 ± 0.12 | 8.6 ± 0.13 | 8.5 ± 0.40 | 10.3 ± 0.15 | <0.001 |
|  | Mean total hospitalization charges (USD) | 76,511 ± 1168 | 101,213 ± 15671 | 77,720 ± 2300 | 87,546 ± 1930 | 87,419 ± 2413 | 86,538 ± 5146 | 127,376 ± 3099 | <0.001 |

# Supplementary Table S6

Outcomes of univariate and multivariable regression adjusted analysis stratified by CKD stages.

|  |  | **Kidney disease-free** | **CKD1** | **CKD2** | **CKD3** | **CKD4** | **CKD5** | **ESKD** |
| --- | --- | --- | --- | --- | --- | --- | --- | --- |
| In hospital mortality | Crude OR (95% CI) | 1.00 | 1.68 (1.14-2.49) | 1.60 (1.40-1.83) | 2.22 (2.08-2.37) | 3.11 (2.88-3.36) | 3.55 (2.84-4.42) | 2.36 (2.22-2.51) |
|  | P value |  | 0.010 | <0.001 | <0.001 | <0.001 | <0.001 | <0.001 |
|  | aOR (95% CI) | 1.00 | 1.41  (0.92-2.16) | 1.10 (0.95-1.28) | **1.34 (1.25-1.44)** | **1.80 (1.66-1.97)** | **2.66 (2.08-3.40)** | **1.97 (1.83-2.12)** |
|  | P value |  | 0.67 | 0.192 | **<0.001** | **<0.001** | **<0.001** | **<0.001** |
| Septic shock | Crude OR (95% CI) | 1.00 | 1.78 (0.95-3.34) | 1.34 (1.08-1.68) | 1.521 (1.34-1.71) | 1.72 (1.48-2.00) | 2.09 (1.39-3.14) | 2.81 (2.56-3.08) |
|  | P value |  | 0.071 | 0.009 | <0.001 | <0.001 | <0.001 | <0.001 |
|  | aOR (95% CI) | 1.00 | 1.73 (0.91-3.29) | 1.04 (0.81-1.35) | **1.22 (1.07-1.39)** | **1.43 (1.33-1.66)** | **1.61 (1.02-2.54)** | **2.12 (1.90-2.37)** |
|  | P value |  | 0.094 | 0.739 | **0.004** | **<0.001** | **0.04** | **<0.001** |
| Acute respiratory failure | Crude OR (95% CI) | 1.00 | 1.10 (0.83-1.45) | 1.02 (0.94-1.11) | 0.98 (0.93-1.03) | 1.02 (0.96-1.09) | 0.80 (0.66-0.97) | 0.94 (0.89-0.98) |
|  | P value |  | 0.526 | 0.599 | 0.429 | 0.489 | 0.026 | 0.007 |
|  | aOR (95% CI) | 1.00 | 1.05 (0.77-1.42) | 0.94 (0.86-1.03) | **0.92 (0.87-0.97)** | 0.94 (0.88-1.01) | 0.82 (0.66-1.01) | **0.94 (0.89-0.99)** |
|  | P value |  | 0.769 | 0.179 | **0.001** | 0.099 | 0.060 | **0.014** |
| ARDS | Crude OR (95% CI) | 1.00 | 1.70 (0.99-2.88) | 1.14 (0.94-1.38) | 1.18 (1.06-1.31) | 1.37 (1.21-1.55) | 1.35 (0.92-1.99) | 1.25 (1.13-1.39) |
|  | P value |  | 0.052 | 0.180 | 0.003 | <0.001 | 0.124 | <0.001 |
|  | aOR (95% CI) | 1.00 | 1.71 (0.99-2.94) | 1.03 (0.84-1.26) | 1.10 (0.98-1.23) | **1.30 (1.14-1.49)** | 1.30 (0.87-1.93) | 1.07 (0.96-1.20) |
|  | P value |  | 0.053 | 0.779 | 0.097 | **<0.001** | 0.200 | 0.233 |
| Mechanical ventilation | Crude OR (95% CI) | 1.00 | 1.61 (1.01-2.58) | 1.49 (1.29-1.72) | 1.42 (1.32-1.54) | 1.67 (1.51-1.85) | 2.01 (1.53-2.65) | 1.72 (1.60-1.85) |
|  | P value |  | 0.046 | <0.001 | <0.001 | <0.001 | <0.001 | <0.001 |
|  | aOR (95% CI) | 1.00 | 1.48 (0.92-2.38) | 1.12 (0.95-1.31) | 1.05 (0.96-1.14) | **1.19 (1.07-1.32)** | **1.56 (1.16-2.08)** | **1.29 (1.19-1.40)** |
|  | P value |  | 0.110 | 0.172 | 0.295 | **0.001** | **0.003** | **<0.001** |
| Vasopressor | Crude OR (95% CI) | 1.00 | 1.29 (0.49-3.44) | 1.51 (1.13-2.02) | 1.55 (1.35-1.79) | 1.55 (1.24-1.93) | 2.61 (1.56-4.36) | 2.51 (2.20-2.86) |
|  | P value |  | 0.608 | 0.006 | <0.001 | <0.001 | <0.001 | <0.001 |
|  | aOR (95% CI) | 1.00 | 1.18 (0.44-3.22) | 1.16 (0.83-1.61) | **1.27 (1.10-1.48)** | **1.29 (1.02-1.64)** | **2.18 (1.27-3.74)** | **1.96 (1.68-2.28)** |
|  | P value |  | 0.741 | 0.390 | **0.002** | **0.032** | **0.005** | **<0.001** |
| LOS | β (95% CI) | Ref | 1.81 (0.30-3.31) | 1.01 (0.67-1.35) | 1.97 (1.75-2.20) | 1.34 (1.08-1.59) | 1.33 (0.55-2.11) | 3.10 (2.82-3.39) |
|  | P value |  | 0.018 | <0.001 | <0.001 | <0.001 | 0.001 | <0.001 |
|  | Adjusted β (95% CI) | Ref | 1.22 (-0.29-2.73) | **0.43 (0.08-0.77)** | **1.11 (0.88-1.34)** | **0.47 (0.22-0.73)** | 0.36 (-0.42-1.15) | **2.31 (2.01-2.61)** |
|  | P value |  | 0.112 | **0.015** | **<0.001** | **<0.001** | 0.366 | **<0.001** |
| Total hospitalization charges | β (95% CI) | Ref | 24,702 (-5980- 55,384) | 1209 (-3410-5827) | 11,035 (7621-14,449) | 10,908 (6492-15,324) | 10,027 (-11.29-20,066) | 50,865 (45,548-56,182) |
|  | P Value |  | 0.115 | 0.608 | <0.001 | <0.001 | 0.050 | <0.001 |
|  | Adjusted β (95% CI) | Ref | 21,549 (-11,257- 54,355) | -1828 (-6598- 2941) | **4604 1051-8158)** | **5551 (1113-9988)** | 2579 (-7950-13109) | **37,268 (31,938-42,598)** |
|  | P Value |  | 0.198 | 0.452 | **0.011** | **0.014** | 0.631 | **<0.001** |

# Supplementary Table S7

Sensitivity analysis with selective confounding variables to identify independent risk factors of mortality in patients admitted with COVID-19

|  | **Univariate regression model** | | **Multivariable regression Model** | |
| --- | --- | --- | --- | --- |
|  | **OR (95% CI)** | **P-value** | **OR (95%)** | **P-value** |
| **Age** | 1.05 (1.04-1.05) | <0.001 | **1.05 (1.05-1.05)** | **<0.001** |
| **Race**  **(White as reference)** | B: 0.83 (0.79-0.88) | <0.001 | **1.07 (1.01-1.13)** | **0.013** |
|  | H: 0.89 (0.84-0.94) | <0.001 | **1.39 (1.31-1.47)** | **<0.001** |
|  | AP: 0.91 (0.82-1.01) | 0.081 | **1.18 (1.06-1.31)** | **0.003** |
|  | NA: 1.33 (1.13-1.56) | 0.001 | **2.02 (1.69-2.41)** | **<0.001** |
| **Obesity** | 0.79 (0.76-0.82) | <0.001 | **1.16 (1.12-1.21)** | **<0.001** |
| **Hypertension** | 1.59 (1.53-1.65) | <0.001 | **0.82 (0.79-0.86)** | **<0.001** |
| **Diabetes** | 1.36 (1.32-1.40) | <0.001 | **1.18 (1.14-1.21)** | **<0.001** |
| **Coronary artery disease** | 1.75 (1.70-1.81) | <0.001 | 1.02 (0.99-1.06) | 0.21 |
| **Chronic heart failure** | 2.36 (2.28-2.44) | <0.001 | **1.51 (1.45-1.57)** | **<0.001** |
| **Chronic lung disease** | 1.23 (1.19-1.27) | <0.001 | **1.07 (1.03-1.11)** | **0.001** |
| **Chronic liver disease** | 2.10 (1.99-2.23) | <0.001 | **2.61 (2.46-2.77)** | **<0.001** |
| **Chronic kidney disease**  **(Compared to no kidney disease)** | CKD1: 1.68 (1.14-2.49) | 0.010 | 1.35 (0.89-2.03) | 0.155 |
|  | CKD2: 1.60 (1.40-1.83) | <0.001 | **1.17 (1.01-1.34)** | **0.034** |
|  | CKD3: 2.22 (2.08-2.37) | <0.001 | **1.36 (1.27-1.46)** | **<0.001** |
|  | CKD4: 3.11 (2.88-3.36) | <0.001 | **1.76 (1.62-1.91)** | **<0.001** |
|  | CKD5: 3.55 (2.84-4.42) | <0.001 | **2.61 (2.05-3.33)** | **<0.001** |
|  | ESKD: 2.36 (2.22-2.51) | <0.001 | **2.03 (1.89-2.18)** | **<0.001** |
|  | KT: 1.38 (1.12-1.70) | 0.002 | **1.70 (1.37-2.10)** | **<0.001** |

# Supplementary Table S8

Sensitivity analysis with selective confounding variables: outcomes of univariate and multivariable regression adjusted analysis stratified by kidney disease.

|  |  | **kidney disease-free** | **Advanced CKD** | **ESKD** | **KT** |
| --- | --- | --- | --- | --- | --- |
| In hospital mortality | OR (95% CI) | 1.00 | 2.54 (2.42-2.68) | 2.36 (2.22-2.51) | 1.38 (1.12-1.70) |
|  | P value |  | <0.001 | <0.001 | 0.002 |
|  | aOR (95% CI) | 1.00 | **1.52 (1.44-1.61)** | **2.03 (1.89-2.17)** | **1.69 (1.37-2.10)** |
|  | P value |  | **<0.001** | **<0.001** | **<0.001** |
| Septic shock | OR (95% CI) | 1.00 | 1.60 (1.45-1.76) | 2.81 (2.56-3.08) | 1.56 (1.13-2.15) |
|  | P value |  | <0.001 | <0.001 | 0.007 |
|  | aOR (95% CI) | 1.00 | **1.39 (1.25-1.55)** | **2.42 (2.18-2.68)** | **1.57 (1.12-2.19)** |
|  | P value |  | **<0.001** | **<0.001** | **0.009** |
| Acute respiratory failure | OR (95% CI) | 1.00 | 0.99 (0.95-1.03) | 0.94 (0.89-0.98) | 0.67 (0.59-0.77) |
|  | P value |  | 0.529 | 0.007 | <0.001 |
|  | aOR (95% CI) | 1.00 | **0.92 (0.88-0.96)** | **0.94 (0.89-0.99)** | **0.73 (0.64-0.84)** |
|  | P value |  | **<0.001** | **0.016** | **<0.001** |
| ARDS | OR (95% CI) | 1.00 | 1.25 (1.15-1.35) | 1.25 (1.13-1.39) | 1.22 (0.92-1.61) |
|  | P value |  | <0.001 | <0.001 | 0.159 |
|  | aOR (95% CI) | 1.00 | **1.21 (1.10-1.32)** | **1.16 (1.04-1.29)** | 1.30 (0.98-1.71) |
|  | P value |  | **<0.001** | **0.008** | 0.07 |
| Mechanical ventilation | OR (95% CI) | 1.00 | 1.52 (1.43-1.62) | 1.72 (1.60-1.85) | 1.02 (0.80-1.30) |
|  | P value |  | <0.001 | <0.001 | 0.865 |
|  | aOR (95% CI) | 1.00 | **1.12 (1.05- 1.20)** | **1.34 (1.24-1.44)** | 1.01 (0.79-1.29) |
|  | P value |  | **0.001** | **<0.001** | 0.938 |
| Vasopressor | OR (95% CI) | 1.00 | 1.59 (1.41-1.79) | 2.51 (2.20-2.86) | 1.11 (0.68-1.79) |
|  | P value |  | <0.001 | <0.001 | 0.681 |
|  | aOR (95% CI) | 1.00 | **1.39 (1.22-1.57)** | **2.05 (1.78-2.37)** | 1.10 (0.67-1.79) |
|  | P value |  | **<0.001** | **<0.001** | 0.701 |
| LOS | β (95% CI) | Ref | 1.74 (1.57-1.92) | 3.10 (2.82-3.39) | -0.19 (-0.69-0.30) |
|  | P value |  | <0.001 | <0.001 | 0.446 |
|  | Adjusted β (95% CI) | Ref | **1.01 (0.83-1.19)** | **2.52 (2.22-2.82)** | -0.10 (-0.59- 0.40) |
|  | P value |  | **<0.001** | **<0.001** | 0.705 |
| Total hospitalization charges | β (95% CI) | Ref | 10,958 (8171-13,745) | 50,865 (45,548-56,182) | 5249 (-3499- 13,997) |
|  | P Value |  | <0.001 | <0.001 | 0.240 |
|  | Adjusted β (95% CI) | Ref | **7431.59 (4635- 10,227)** | **41,243 (35,822-46,664)** | 4079 (-4581- 12378) |
|  | P Value |  | **<0.001** | **<0.001** | 0.356 |

ARDS: acute respiratory distress syndrome; LOS: length of hospital stay; CI: Confidence interval; aOR: adjusted odds ratio

# Supplementary Table S9

Sensitivity analysis with selective confounding variables: outcomes of univariate and multivariable regression adjusted analysis stratified by CKD stages.

|  |  | **Kidney disease-free** | **CKD1** | **CKD2** | **CKD3** | **CKD4** | **CKD5** | **ESKD** |
| --- | --- | --- | --- | --- | --- | --- | --- | --- |
| In hospital mortality | Crude OR (95% CI) | 1.00 | 1.68 (1.14-2.49) | 1.60 (1.40-1.83) | 2.22 (2.08-2.37) | 3.11 (2.88-3.36) | 3.55 (2.84-4.42) | 2.36 (2.22-2.51) |
|  | P value |  | 0.010 | <0.001 | <0.001 | <0.001 | <0.001 | <0.001 |
|  | aOR (95% CI) | 1.00 | 1.35 (0.89-2.03) | **1.17 (1.01-1.34)** | **1.36 (1.27-1.46)** | **1.76 (1.62-1.91)** | **2.61 (2.05-3.33)** | **2.03 (1.89-2.18)** |
|  | P value |  | 0.16 | **0.034** | **<0.001** | **<0.001** | **<0.001** | **<0.001** |
| Septic shock | Crude OR (95% CI) | 1.00 | 1.78 (0.95-3.34) | 1.34 (1.08-1.68) | 1.521 (1.34-1.71) | 1.72 (1.48-2.00) | 2.09 (1.39-3.14) | 2.81 (2.56-3.08) |
|  | P value |  | 0.071 | 0.009 | <0.001 | <0.001 | <0.001 | <0.001 |
|  | aOR (95% CI) | 1.00 | 1.72 (0.92-3.22) | 1.22 (0.96-1.55) | **1.33 (1.17-1.52)** | **1.45 (1.25-1.69)** | **1.84 (1.20-2.83)** | **2.42 (2.18-2.68)** |
|  | P value |  | 0.088 | 0.104 | **<0.001** | **<0.001** | **0.006** | **<0.001** |
| Acute respiratory failure | Crude OR (95% CI) | 1.00 | 1.10 (0.83-1.45) | 1.02 (0.94-1.11) | 0.98 (0.93-1.03) | 1.02 (0.96-1.09) | 0.80 (0.66-0.97) | 0.94 (0.89-0.98) |
|  | P value |  | 0.526 | 0.599 | 0.429 | 0.489 | 0.026 | 0.007 |
|  | aOR (95% CI) | 1.00 | 1.03 (0.76-1.38) | 0.96 (0.88-1.05) | **0.93 (0.88-0.98)** | **0.93 (0.87-1.00)** | **0.77 (0.63-0.94)** | **0.94 (0.89-0.99)** |
|  | P value |  | 0.862 | 0.385 | **0.004** | **0.039** | **0.011** | **0.015** |
| ARDS | Crude OR (95% CI) | 1.00 | 1.70 (0.99-2.88) | 1.14 (0.94-1.38) | 1.18 (1.06-1.31) | 1.37 (1.21-1.55) | 1.35 (0.92-1.99) | 1.25 (1.13-1.39) |
|  | P value |  | 0.052 | 0.180 | 0.003 | <0.001 | 0.124 | <0.001 |
|  | aOR (95% CI) | 1.00 | 1.71 (1.0-2.93) | 1.13 (0.93-1.38) | **1.14 (1.02-1.28**) | **1.31 (1.15-1.50)** | 1.35 (0.92-1.99) | **1.06 (1.04-1.29**) |
|  | P value |  | 0.052 | 0.210 | **0.020** | **<0.001** | 0.123 | **0.007** |
| Mechanical ventilation | Crude OR (95% CI) | 1.00 | 1.61 (1.01-2.58) | 1.49 (1.29-1.72) | 1.42 (1.32-1.54) | 1.67 (1.51-1.85) | 2.01 (1.53-2.65) | 1.72 (1.60-1.85) |
|  | P value |  | 0.046 | <0.001 | <0.001 | <0.001 | <0.001 | <0.001 |
|  | aOR (95% CI) | 1.00 | 1.37 (0.83-2.19) | **1.20 (1.03-1.40)** | 1.07 (0.98-1.16) | **1.17 (1.06-1.30)** | **1.55 (1.17-2.06)** | **1.34 (1.24-1.44)** |
|  | P value |  | 0.194 | **0.018** | 0.127 | **0.003** | **0.003** | **<0.001** |
| Vasopressor | Crude OR (95% CI) | 1.00 | 1.29 (0.49-3.44) | 1.51 (1.13-2.02) | 1.55 (1.35-1.79) | 1.55 (1.24-1.93) | 2.61 (1.56-4.36) | 2.51 (2.20-2.86) |
|  | P value |  | 0.608 | 0.006 | <0.001 | <0.001 | <0.001 | <0.001 |
|  | aOR (95% CI) | 1.00 | 1.21 (0.45-3.25) | **1.38 (1.02-1.88)** | **1.37 (1.18-1.58)** | **1.33 (1.05-1.67)** | **2.34 (1.40-3.92)** | **2.05 (1.78-2.37)** |
|  | P value |  | 0.701 | **0.037** | **<0.001** | **0.016** | **0.001** | **<0.001** |
| LOS | β (95% CI) | Ref | 1.81 (0.30-3.31) | 1.01 (0.67-1.35) | 1.97 (1.75-2.20) | 1.34 (1.08-1.59) | 1.33 (0.55-2.11) | 3.10 (2.82-3.39) |
|  | P value |  | 0.018 | <0.001 | <0.001 | <0.001 | 0.001 | <0.001 |
|  | Adjusted β (95% CI) | Ref | 1.13 (-0.32-2.58) | **0.55 (0.21-0.88)** | **1.26 (1.04-1.49)** | **0.54 (0.28-0.80)** | 0.62 (-0.16-1.39) | **2.52 (2.22-2.82)** |
|  | P value |  | 0.125 | **0.002** | **<0.001** | **<0.001** | 0.119 | **<0.001** |
| Total hospitalization charges | β (95% CI) | Ref | 24,702 (-5980- 55,384) | 1209 (-3410-5827) | 11,035 (7621-14,449) | 10,908 (6492-15,324) | 10,027 (-11.29-20,066) | 50,865 (45,548-56,182) |
|  | P Value |  | 0.115 | 0.608 | <0.001 | <0.001 | 0.050 | <0.001 |
|  | Adjusted β (95% CI) | Ref | 22,356 (-8,520- 53,230) | 501 (-4155- 5158) | **8102 (4,700-11,504)** | **6239 (1,831-10,648)** | 5,888 (-4132-15,908) | **41,235 (35,813-46,656)** |
|  | P Value |  | 0.156 | 0.833 | **<0.001** | **<0.001** | 0.249 | **<0.001** |
